# Interaction Between Conservation Tillage and Nitrogen Fertilization Shapes Prokaryotic and Fungal Diversity at Different Soil Depths: Evidence From a 23-Year Field Experiment in the Mediterranean Area

**DOI:** 10.3389/fmicb.2019.02047

**Published:** 2019-09-04

**Authors:** Gaia Piazza, Laura Ercoli, Marco Nuti, Elisa Pellegrino

**Affiliations:** Institute of Life Sciences, Scuola Superiore Sant’Anna, Pisa, Italy

**Keywords:** soil bacteria, soil fungi, soil archaea, arbuscular mycorrhizal fungi, Glomeromycota, Illumina sequencing, 16S rRNA, ITS1 region

## Abstract

Soil biodiversity accomplishes key roles in agro-ecosystem services consisting in preserving and enhancing soil fertility and nutrient cycling, crop productivity and environmental protection. Thus, the improvement of knowledge on the effect of conservation practices, related to tillage and N fertilization, on soil microbial communities is critical to better understand the role and function of microorganisms in regulating agro-ecosystems. In the Mediterranean area, vulnerable to climate change and suffering for management-induced losses of soil fertility, the impact of conservation practices on soil microbial communities is of special interest for building mitigation and adaptation strategies to climate change. A long-term experiment, originally designed to investigate the effect of tillage and N fertilization on crop yield and soil organic carbon, was utilized to understand the effect of these management practices on soil prokaryotic and fungal community diversity. The majority of prokaryotic and fungal taxa were common to all treatments at both soil depths, whereas few bacterial taxa (Cloacimonates, *Spirochaetia* and Berkelbacteria) and a larger number of fungal taxa (i.e., *Coniphoraceae*, *Debaryomycetaceae*, *Geastraceae*, *Cordicypitaceae* and *Steccherinaceae*) were unique to specific management practices. Soil prokaryotic and fungal structure was heavily influenced by the interaction of tillage and N fertilization: the prokaryotic community structure of the fertilized conventional tillage system was remarkably different respect to the unfertilized conservation and conventional systems in the surface layer. In addition, the effect of N fertilization in shaping the fungal community structure of the surface layer was higher under conservation tillage systems than under conventional tillage systems. Soil microbial community was shaped by soil depth irrespective of the effect of plowing and N addition. Finally, chemical and enzymatic parameters of soil and crop yields were significantly related to fungal community structure along the soil profile. The findings of this study gave new insights on the identification of management practices supporting and suppressing beneficial and detrimental taxa, respectively. This highlights the importance of managing soil microbial diversity through agro-ecological intensified systems in the Mediterranean area.

## Introduction

Agricultural systems are essential sources of provision services, such as food, forage, fiber, and bioenergy, as well as for regulating services, such as climate and disease regulation (Power, 2010). High intensity of management practices has potential negative environmental consequences, which may determine the reduction of agricultural incomes and the increase of production costs (Zhang et al., 2007). Intensive plow-based systems along with inefficient use of chemical inputs are known to accelerate the oxidation of soil organic matter (SOM), disrupt soil physical structure, decrease soil moisture retention and modify soil biological diversity, with strong consequences on biogeochemical cycles (Peng et al., 2013; Degrune et al., 2017; Wang et al., 2018). Moreover, agricultural activities have been recognized responsible for 30% of global anthropogenic greenhouse gas emissions that are one of the causes of global warming and climate change (IPCC, 2015).

Conservation agriculture (CA) is a systemic approach aiming to a sustainable intensification of production, coping with the ecosystem disservices determined by conventional management practices (Pretty, 2008; Friedrich, 2013). Conservation agriculture is based on the application of the following linked principles: minimum or no soil disturbance; maintenance of permanent soil mulch cover; adoption of crop rotations and an appropriate use of fertilizers (Lal, 2015; Thierfelder et al., 2018). Conservation agriculture covers worldwide about 180 million ha across different agro-ecosystems, representing 13% of the global cropland, with an increase of about 69% from 2015–2016 to 2008–2009 (Kassam et al., 2019).

Soil biodiversity accomplishes key roles in ecosystem services, consisting in preserving and enhancing soil fertility and nutrient cycling, crop productivity and environmental protection (Yang et al., 2017; Mommer et al., 2018; Wang et al., 2019). Thus, the improvement of knowledge on patterns and drivers of soil microbial community composition is critical to better understand the role and function of soil microorganisms in regulating agro-ecosystem structure, process and functioning. Linkages between above- and belowground communities of living organisms have been largely investigated: plant community composition can shape the diversity of belowground organisms, such as decomposers, symbionts or pathogens, whose activity affect plant metabolism and shoot/root biomass production (Bezemer and van Dam, 2005; De Deyn and Van der Putten, 2005; Bardgett and Wardle, 2010; van Der Heijden et al., 2016). In this context, bacteria and fungi represent the dominant microorganisms in soil, consisting in 10^2^–10^4^ μg biomass carbon (C) g^–1^ soil (Fierer, 2017) and carrying out 80–90% of soil processes (Nannipieri and Badalucco, 2003).

Several studies found increases of microbial biomass under long-term conservation tillage systems (i.e., no tillage, NT; minimum tillage, MT) (e.g., Mathew et al., 2012; Zhang et al., 2012; Murugan et al., 2014). Moreover, recent studies, addressing the molecular diversity of microbial communities, have shown a higher abundance of Acidobacteria and Bacteroidetes and a lower abundance of Proteobacteria and Actinobacteria under MT systems respect to conventional tillage (CT) (Dorr de Quadros et al., 2012; Degrune et al., 2017; Babin et al., 2019). These evidences were explained through the different nutrient status and aerobic/anaerobic conditions of soil. According to the oligotrophy-copiotrophy theory, fast-growing r-strategist microorganisms (or copiotrophics, e.g., classes *Alpha*- and *Beta-proteobacteria*), showing a preference to use labile C compounds, are highly present in CT (Fontaine et al., 2003; Fierer et al., 2007; Trivedi et al., 2015, 2017). Conversely, slow-growing K-strategists (or oligotrophics, e.g., phylum Acidobacteria), mainly feeding on recalcitrant C, are highly present in MT. Moreover, under NT or MT, a shift from soil bacterial- to fungal-dominated communities was associated to SOC accumulation, due to the improvement of soil structural stability through mycelium development and deposition of fungal-derived C (Simpson et al., 2004; Kong et al., 2011). Minimum soil disturbance could also allow suitable conditions for the promotion of the abundance and diversity of symbiotic fungi, such as arbuscular mycorrhizal fungi (AMF), as well as shifts of their community structures (Alguacil et al., 2008; Brito et al., 2012). Arbuscular mycorrhizal fungi can improve soil aggregation and structure on one side, and enhance plant growth, crop production, plant nutrient status and health on the other side (Ercoli et al., 2017; Caruso, 2018; Powell and Rillig, 2018; Zhang et al., 2018; Coccina et al., 2019). However, according to a recent meta-analysis, the response of soil microbial diversity to tillage is highly variable depending on climatic conditions and soil properties (de Graaff et al., 2019). Overall, MT systems increase bacterial diversity by 7% and do not affect either soil total fungal diversity or AMF diversity respect to CT. The Mediterranean area is characterized by summer drought and erratic distribution of rainfall and thus is vulnerable to climate change and suffers for losses of soil fertility. In these conditions, the strong decrease of AMF diversity following CT and the large difference in the responses of functional bacterial and fungal taxa need to be better clarified (Ceja-Navarro et al., 2010; Brito et al., 2012; Pastorelli et al., 2013; Ciccolini et al., 2015, 2016a,b; Pellegrino et al., 2015b).

Bacterial diversity was increased by mineral N fertilization at low input (<150 kg N ha^–1^ y^–1^) and with applications over more than 5 years, while fungal diversity was increased only for specific taxonomic groups (e.g., yeasts), having a copiotrophic lifestyle (de Graaff et al., 2019). On the other hand, AMF diversity was reduced by N fertilization and this was supposedly due to a less investment in mycorrhizal symbioses by host plants (Treseder, 2004) and to a reduction in pH induced by N addition that improves phosphorus (P) availability (Egerton-Warburton et al., 2007; Marklein and Houlton, 2012). In the Mediterranean cropping systems, also suffering from leaching N and S losses, the effect of N fertilization on the diversity and structure of functional soil prokaryotes and fungi was poorly investigated (Dias et al., 2000; Ercoli et al., 2012; Alguacil et al., 2014; Mariotti et al., 2015). Therefore, in this area the impact of agricultural management (i.e., tillage and N fertilization) on soil microbial communities needs to be better clarified in order to reduce environmental pollution and water deficit and improve crop yield and SOC accumulation.

Gradient of soil nutrients and environmental factors throughout the soil profile have been shown to affect abundance, composition and structure of soil prokaryotic and fungal communities (Fierer et al., 2003; Oehl et al., 2005; Eilers et al., 2012; Somenahally et al., 2018; Yao et al., 2018). In the surface layer, where the availability of nutrients is high, bacteria prevail over fungi, whereas in the lower layer the opposite occurs, and fungi prevail since they are more competitive for the uptake of N and P. By contrast, if a large amount of plant material input and recalcitrant organic matter is in the surface layer, the fungal biomass prevails over bacteria, since fungi are able to decompose more complex organic matter (Jumpponen et al., 2010). Therefore, there is still a need to clarify the composition and structure of soil microbial community shifts along the soil depth profile.

Long-term experiments in the Mediterranean area, such as the one located in Pisa (Italy) and utilized for this study, originally designed to investigate the effect of tillage and N fertilization on crop yield and SOC, provide a great opportunity for improving our understanding on the effect of management practices on soil prokaryotic and fungal diversity. In this study, we tested the following hypotheses: (1) the interaction of long-term conservation tillage and N fertilization shifts molecular community diversity, composition and structure of soil prokaryotes and fungi; (2) soil depth plays a role in shaping the diversity, composition and structure of soil prokaryotes and fungi; (3) soil physico-chemical and biochemical parameters affect soil microbial community structure and in reverse microbes affect soil parameters, with specific taxa playing major roles in nutrient cycling.

## Materials and Methods

### Field Experiment

The long-term field experiment was set up in 1993 comparing two tillage intensities to a bread wheat (*Triticum aestivum* L.) - soybean (*Glycine max* L. Merr.) rotation: conventional tillage (CT), mouldboard plowing at 25-cm depth, disking and harrowing at 15-cm depth; MT, disk harrowing at 15-cm depth. Two N fertilization levels were also applied on bread wheat: 0 and 200 kg N ha^–1^ (N0 and N200, respectively). These treatments were applied arranged following a split-plot design with tillage as main-plot factor and N fertilization as subplot factor, with three replicate plots (dimension: 11.5 × 14.5 m). The experiment was conducted at the Centro Interdipartimentale di Ricerche Agro-Ambientali “Enrico Avanzi” (San Piero a Grado, Pisa, Italy; 43°40’ latitude N; 10°19’ longitude E; 1 m above sea level) of the University of Pisa in an alluvial silt loam soil (131, 613 and 256 g kg^–1^ of sand, silt and clay, respectively, in the 0–30 cm soil layer). The soil is classified as Typic Xerofluvent by USDA system (Soil Survey Staff, 1975) and as Fluvisol by FAO (IUSS working group WRB, 2006). Climate of the site is cold, humid Mediterranean (Csa), according to the Köppen–Geiger climate classification (Kottek et al., 2006). Nitrogen fertilizer treatment was applied to wheat as urea and the rate was splitted into three applications before seeding (60 kg N ha^–1^), at first node detectable (70 kg N ha^–1^), and 15 days after this stage (70 kg N ha^–1^). Under CT, almost 100% of the residues were incorporated in the 0–25 cm soil layer, whereas under MT approximately 50% of the crop residues were incorporated at 0–15-cm depth. Crops were managed following the common agronomical technique applied in the area, comprising pre-emergence herbicide application for weed control, whereas no disease or insect treatment was applied.

### Soil Sampling and Analysis of Soil Physico-Chemical and Enzymatic Parameters

For the evaluation of stable systems after long-term changes, soil samples were collected in Spring 2016, before soybean sowing in order to avoid sampling close to the main management practices (Picci and Nannipieri, 2002; Pellegrino et al., 2011). Spring is considered the best time to assess soil microbial diversity in the study area, since the land is dry enough to access and soil temperature is optimal for the growth of microbes. This depends to the fact that in the area the risk of high and low rainfall events is the lowest in Spring compared with other seasons and air temperature does not show low and high extreme values (Vallebona et al., 2015). In each replicate plot, a homogenized sample was taken by mixing the content of four soil cores collected at two soil depths (0–15 and 15–30 cm). Once in the laboratory, each sample was air-dried, gently broken apart and then passed through a 2-mm sieve.

Soil samples were analyzed for soil bulk density (BD), soil organic carbon (SOC), total nitrogen (Total N), available P (Avail P), ammonium (NH_4_-N) and nitrate (NO_3_-N). Moreover, soil enzyme potential activities were measured as: cellulose (Cell), chitinase (NAG), α-glucosidase (α-gluc), β-glucosidase (β-gluc), xylosidase (Xylos), phosphatase (Phosph), arylsulphatase (Aryls) and leucine (L.AP). The synthetic enzymatic index (SEI), synthetic enzyme index for the C-cycle (SEIc) were calculated, as well as the microbial functional diversity (Shannon diversity index, *H*). Finally, the ecoenzymatic C/N and N/P acquisition activities were measured by the ratios of β-glucosidase/(chitinase + leucine) [β-gluc/(NAG + L.AP)] and (chitinase + leucine)/phosphatase activities [(NAG + L.AP)/Phosph], respectively. Details about the analytical methods are given in Supplementary Materials and Methods.

### Molecular Analyses

DNA was extracted from 0.25 g of soil samples using the DNeasy PowerSoil Kit (QIAGEN, Venlo, Netherlands), following the instructions of the manufacturer. The extracted DNA was quantified by a spectrophotometer (NanoDrop Technology, Wilmington, DE) and then stored at −20°C for further analyses. PCRs were generated from 10 ng μL^–1^ genomic DNA in volumes of 25 μL with 0.125 U μL^–1^ of GoTaq^®^ Hot Start Polymerase (Promega Corporation, WA, United States), 0.5 μM of each primer set for prokaryotes and fungi, 0.2 mM of each dNTP, 1 mM of MgCl_2_ and 1x reaction buffer, using a S1000 Thermal Cycler^TM^ (BioRad, Hercules, CA, United States). The primer sets used for the amplification of prokaryotes and fungi, relative sequences, targeted genes and PCR reaction conditions are given in Supplementary Table S1. All PCR amplifications were carried out using the primer pairs that have Illumina sequencing tags attached, and in the case of the forward primers a 13 bp random sequence was included to improve cluster definition on the MiSeq slide. All PCR products were examined by electrophoresis through a 1% agarose gel in 0.5 × TBE buffer, then purified with magnetic beads (Agencourt^®^ AMPure^®^ XP, Beckman Coulter, United States) and freshly prepared 80% ethanol, and quantified by a fluorimetry with the Quant-iT^TM^ dsDNA High-Sensitivity Assay Kit (Invitrogen by Thermo Fisher Scientific, CA, United States), following the instructions of the manufacturer. Cleaned and quantified amplicons of each library were adjusted in an equimolar ratio (10 ng/μL) for dual-index barcodes addition using Nextera^®^ Index kit (Illumina Inc., CA, United States), and the resulting metabarcoding libraries were sequenced on an Illumina MiSeq sequencer (2 × 300 bp paired-end reads) at the Eurofins MWG Operon, Ebersberg (Germany).

### Bioinformatic and Statistical Analyses

Raw data generated from the Illumina MiSeq sequencing run were processed and analyzed following the pipelines of QIIME 2 (2018.4) and USEARCH (v10.0.240) (Caporaso et al., 2010; Edgar, 2010). Forward and reverse paired-end sequences were assembled independently for each sample using *-fastq_mergepairs* USEARCH command. Primer sequences were then trimmed off by employing *cutadapt* plugin (2018.4) with default settings. After optimizing the sequences, there were 1,417,762 and 1,173,836 valid sequences for prokaryotes and fungi, respectively. The average length distribution was approximately 260 and 290 for prokaryotes and fungi, respectively. To avoid potential errors in sequencing data, quality of sequence reads was checked by *-fastq_eestats2* USEARCH command, using the expected number of errors in a read as a measure of quality for filtering (Edgar and Flyvbjerg, 2015). Reads were then trimmed at the length where the “drop-off” point for the maximum expected error value occurred (250 bp). Quality filtered reads were de-replicated by *-fastx_uniques* USEARCH command, then operational taxonomic units (OTUs) of prokaryotes and fungi were generated using USEARCH by clustering sequence reads at the 97% similarity threshold. Chimeric sequences and singletons were removed from the dataset during the process.

The OTUs were phylogenetically assigned using two distinct sequence reference databases: the taxonomic identity of prokaryotes was identified by the 16S SSU SILVA database by clustering sequence reads at the 97% similarity threshold (version 132, release date 13.12.2017; Quast et al., 2012; Yilmaz et al., 2013), whereas the identity of fungi by the ITS UNITE database and a dynamic threshold values of clustering (version 7.2, release date 01.12.2017; Kõljalg et al., 2013). For the curation, the sequences were aligned using the algorithms ClustalW and MUSCLE for prokaryotes and fungi, respectively. Neighbor Joining (NJ) phylogenetic trees were built in MEGA7^1^ (Kumar et al., 2016) and then the most abundant sequence of each prokaryotic and fungal OTU was selected, after branch collapsing, and used as representative sequence for that OTU. Then, the representative sequences were re-aligned using the same algorithms and phylogenetic trees were inferred in MEGA7 using the NJ analysis with 1,000 boostrap replicates and the Kimura 2-parameter model (uniform rates) for prokaryotes, and using the maximum likelihood (ML) phylogenetic analysis with 1,000 boostrap replicates and the general time reverse (GTR) evolutionary model (gamma distributed) for Ascomycota, Basidiomycota, Chytridiomycota and the phyla of Glomeromycota and Mortierellomycota, separately. The phylograms were drawn by the interactive tree of life (ITOL) (Letunic and Bork, 2006) and edited by Adobe Illustrator CS4. All representative sequences were deposited in the NCBI sequence read (SRA) database (prokaryotic accession numbers MK903871-MK904485; fungal accession numbers MK881790-MK881896).

Because there was a high variability in the number of reads per sample, sequencing depth per sample was standardized to the median number of reads across the samples in each data matrix (prokaryotes and fungi) using the package Vegan in R (de Cárcer et al., 2011; Oksanen et al., 2013). Applying this approach, bias due to differences in sample size is reduced by randomly choosing in each sample a number of reads equal to the median number of reads across all samples. Samples that had fewer reads than the median were left unchanged. These reads were used for data input in ITOL for building the pie charts, describing the community structure of Prokaryota, Ascomycota and Basidiomycota among treatments at 0–15 and 15–30 cm soil depth.

For testing the hypothesis 1, for each soil depth, richness and diversity indexes (Shannon index, *H’* and Simpson index, λ) were calculated at phylum level and class/family levels (class level: for prokaryotes; family level: for fungi), while the relative abundances for prokaryotes and fungi were calculated at phylum level, and the relative abundances of Ascomycota and Basidiomycota at class level. Richness and diversity indexes were analyzed by a two-way ANOVA following the split-plot experimental design. Data were ln- and arcsine-transformed when needed to fulfill the assumptions of the ANOVA. *Post hoc* Tukey-B significant difference test was used for comparisons among treatments. Means and standard errors given are for untransformed data. All the analyses were performed using the SPSS software package version 21.0 (SPSS Inc., Chicago, IL, United States). Then, the permutational analysis of variance (PERMANOVA; Anderson, 2001) was used to test the effect of tillage (MT and CT) and N fertilization (N0 and N200) on the community structure (relative abundance) of prokaryotes and fungi at phylum and at class/family levels. Response data matrices were square-root transformed prior to the analyses in order to down-weight the importance of dominant taxa and the Bray-Curtis index of dissimilarity was calculated to measure ecological distance. *P*-values were calculated using the Monte-Carlo test (Anderson and Braak, 2003). Since PERMANOVA is sensitive to differences in multivariate location (average community composition of a group) and dispersion (within-group variability), the analysis of homogeneity of multivariate dispersion (PERMDISP; Anderson, 2006) was performed to check the homogeneity of dispersion among groups (beta-diversity) (Anderson et al., 2006). When PERMANOVA indicated a significant effect, the principal coordinate analysis (PCO) was performed (Torgerson, 1958) for visualizing the most relevant patterns in the data. In each PCO biplot, we displayed only the phyla, classes and families with a strong correlation (*r* ≥ 0.70) with the ordination scores indicated on each PCO axis. The circle in each plot, whose diameter is 1.0, allows the reader to understand the scale of the vectors in the vector plot. Analyses were performed using PRIMER 6 and PERMANOVA + software (Clarke and Gorley, 2006; Anderson et al., 2008).

The standardized datasets were also used to generate the Venn diagrams, representing the community composition and unique OTUs to each tillage system and N fertilization or shared among treatments for each soil depth. The Venn diagrams were generated using Venny version 2.1 software^2^ (Oliveros, 2007).

For testing the hypothesis 2, richness and diversity indexes were analyzed by one-way ANOVA using soil depth as fixed factor, and tillage and N fertilization as covariates. Then, the PERMANOVA was used to test the effect of soil depth on the community structure (relative abundances) of prokaryotes and fungi at phylum and at class/family levels. Finally, the standardized datasets were used to generate the Venn diagrams at each soil depth. PERMDISPs and the PCOs were also performed in order to visualize the most relevant patterns and only the phyla, classes and families with a strong correlation (*r* ≥ 0.60) were displayed.

We applied co-inertia analysis (Dolédec and Chessel, 1994) to test the relationship between soil physico-chemical and enzymatic parameters and prokaryotic and fungal community structures (hypothesis 3). The co-inertia approach was utilized because it lacks assumptions on the metrics of the datasets. The first step of the co-inertia analysis was based on a detrended correspondence analysis on the soil microbial community structure using as supplementary variables soil parameters, whereas the second step was based on a principal component analysis on soil parameters. Since co-inertia analysis does not allow testing the significance of the relationship, the analysis was combined with the Mantel test (Mantel and Valand, 1970). Co-inertia analyses can handle a large number of species/variables in both dataset, and no dataset takes the response or predictor role. Co-inertia analyses were performed by CANOCO 5 (ter Braak and Šmilauer, 2012), whereas Mantel test was performed by PC-ORD 5 (Grandin, 2006).

## Results

### Illumina Sequencing Information

**FIGURE 1:**
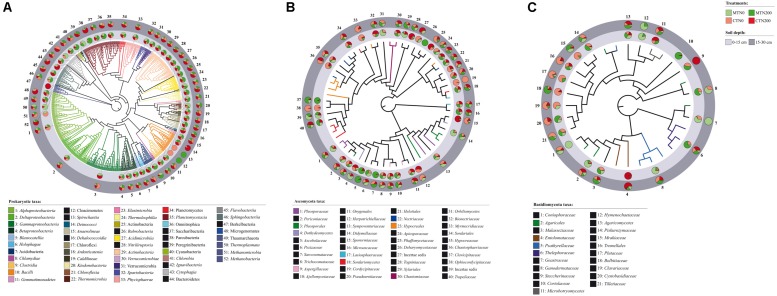
Neighbor-joining (NJ) tree of 52 prokaryotic taxon representative sequences **(A)** and Maximum Likelihood (ML) trees of 40 Ascomycota **(B)** and 21 Basidiomycota taxon representative sequences **(C)** found in soil under long-term tillage and nitrogen fertilization. Treatments are: MTN0 (minimum tillage and 0 kg N ha^–1^), MTN200 (minimum tillage and 200 kg N ha^–1^), CTN0 (conventional tillage and 0 kg N ha^–1^) and CTN200 (conventional tillage and 200 kg N ha^–1^). Soil depths are: 0–15 and 15–30 cm soil depths. NJ tree of prokaryotes is based on the sequences obtained from the amplification of the V4 region (16 SSU rRNA gene) and ML trees of fungi are based on the sequences obtained from the amplification of the ITS1 region (for details see Supplementary Table S1). The prokaryotic taxa were assigned to Operational Taxonomic Unit (OTU) by BLAST against the 16S SSU SILVA database by clustering sequence reads at the 97% similarity threshold. The fungal taxa were assigned by BLAST against the ITS UNITE database and were selected by dynamic threshold values of clustering. For each OTU, the proportion of sequences retrieved from treatment (MTN0, light green; MTN200, dark green; CTN0, light red; CTN200, dark red) and soil depths (0–15 cm: light gray; 15–30 cm: dark gray) are shown in the pie charts. The name of each OTU is composed by the name of the class/phylum phylogenetic resolution and a serial number that is reported also in the trees.

**FIGURE 2:**
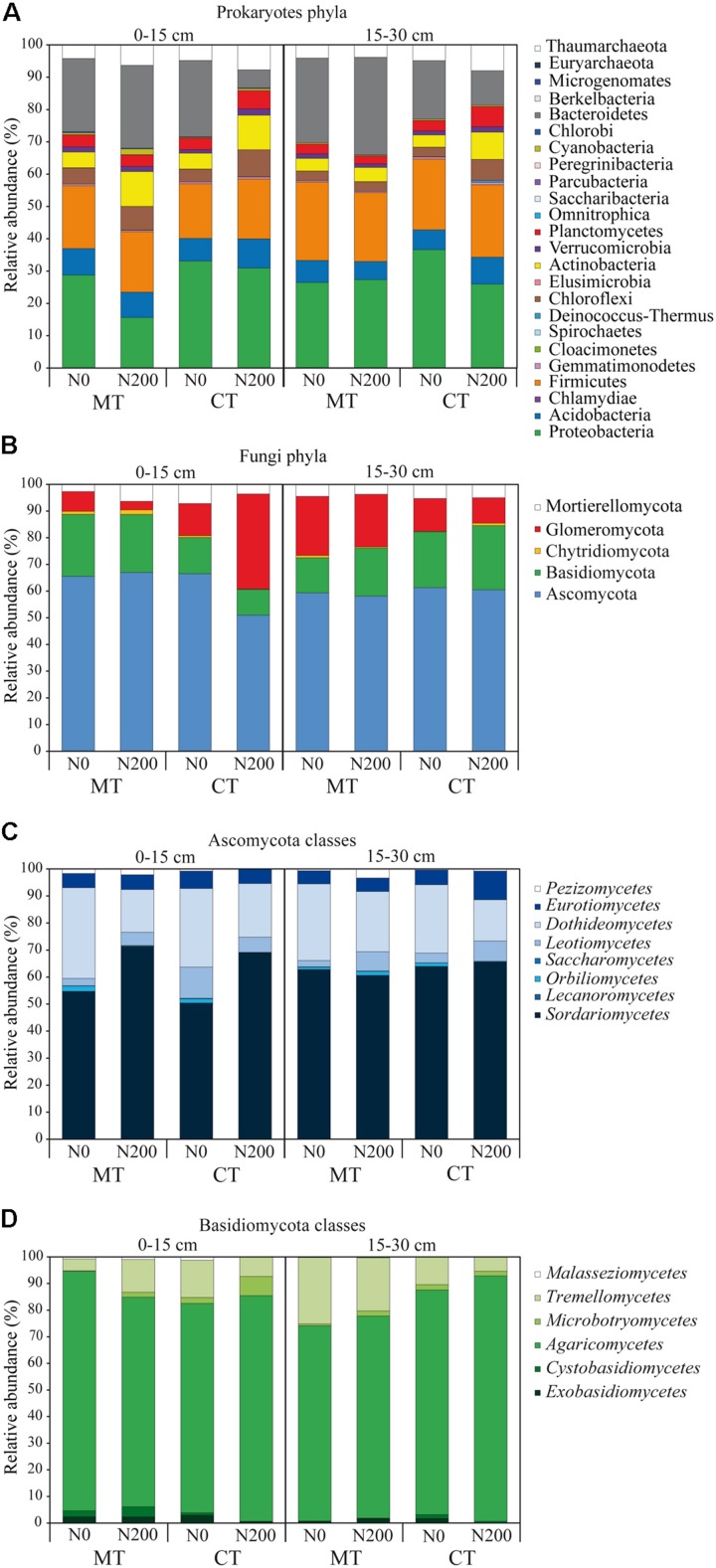
Community diversity of prokaryotic phyla **(A)**, fungal phyla **(B),** Ascomycota classes **(C)**, Basidiomycota classes **(D)** shown as relative abundances of operational taxonomic units found in soil under long-term tillage and nitrogen fertilization. Treatments are: MTN0 (minimum tillage and 0 kg N ha^–1^), MTN200 (minimum tillage and 200 kg N ha^–1^), CTN0 (conventional tillage and 0 kg N ha^–1^) and CTN200 (conventional tillage and 200 kg N ha^–1^). Soil depths are: 0–15 and 15–30 cm soil depths.

A total of 1,341,713 and 1,100,704 quality-filtered 16S SSU rRNA and ITS1 sequences were obtained for prokaryotes and fungi, respectively, from 24 soil samples. For prokaryotes, after BLAST against the 16S SSU SILVA database, we found 1,189,331 reads, ranging from 10 to 103,317 reads per sample that were assigned to a total of 2,875 OTUs. For fungi, after BLAST against the ITS UNITE database we found 1,065,465 reads, ranging from 8 to 174,386 reads per sample that were assigned to a total of 590 OTUs. After the curation of prokaryotic sequences, 1,168,953 reads, ranging from 10 to 94,874 reads per sample, were retrieved and assigned to 615 OTUs (Figure 1A). After the curation of fungal sequences, 1,023,727 reads, ranging from to 8 to 164,728 reads per sample, were retrieved and assigned to 107 OTUs (Figures 1B,C and Supplementary Table S2). Following the standardization to the median number per sample, a total of 1,165,667 reads belonging to 615 OTUs, 52 classes and 24 phyla were retrieved for prokaryotes (Figures 1A, 2A), while for the fungal data, a total of 830,329 reads belonging to 107 OTUs, 68 families, 8 and 6 classes for Ascomycota and Basidiomycota, respectively, were retrieved (Figures 1B,C,2B–D and Supplementary Table S2). Overall, five fungal phyla were retrieved: Ascomycota, Basidiomycota, Chytridiomycota, Glomeromycota and Mortierellomycota (Figure 2B).

### Long-Term Effect of Conservation Tillage and N Fertilization on Community Diversity, Composition and Structure of Soil Prokaryotes (Hypothesis 1)

Prokaryotic richness at class and phylum level at 0–15 cm and at class level at 15–30 cm were not modified either by tillage or N fertilization, whereas at phylum level at 15–30 cm it was modified by tillage (MT < CT: 17.34 *vs.* 19.34 number of OTUs) (Supplementary Table S3). Shannon and Simpson indexes (*H’* and λ, respectively) were significantly increased by N fertilization at both class and phylum level at 0–15 cm (N200 > N0), whereas at 15–30 cm only *H’* at phylum level was increased by tillage (CT > MT).

The total number of prokaryotic classes at 0–15 cm was 47, 44, 49 and 47 under MTN0, MTN200, CTN0 and CTN200, respectively, while at 15–30 was 47, 48, 48 and 49, respectively (Supplementary Figures S1A,B). Looking at a higher phylogenetic resolution level, the total number of prokaryotic phyla at 0–15 cm was 20, 20, 23, 21 under MTN0, MTN200, CTN0 and CTN200, respectively, while at 15–30 was 21 for all treatments (Supplementary Figures S1D,E). Proteobacteria (28%), Bacteroidetes (20%), Firmicutes (20%), Acidobacteria (7%), Actinobacteria (6%), Thaumarchaeota (5%), Chloroflexi (5%), Plantomycetes (4%), Verrucomicrobia (2%), were the dominant phyla, whereas the other phyla occurred with relative abundances below 1% (Figure 2A). Although the majority of those taxa were common to all treatments at both soil depths (0–15 cm: 43 classes and 19 phyla; 15–30 cm: 41 classes and 19 phyla), some were exclusively found in each treatment (Supplementary Figures S1A,B,D,E). Specifically, at 0–15 cm soil depth, the phylum Cloacimonetes (#12) and the class *Spirochaetia* (#13) within the phylum Spirochaetes were retrieved in CTN0, whereas the phylum Berkelbacteria (#47) was retrieved in CTN200 (Figure 1A). By contrast, at 15–30 cm soil depth, the phylum Cloacimonetes and the class *Spirochaetia* were exclusively retrieved in MTN200. However, these unique taxa occurred at very low relative abundances (data not shown).

**FIGURE 3:**
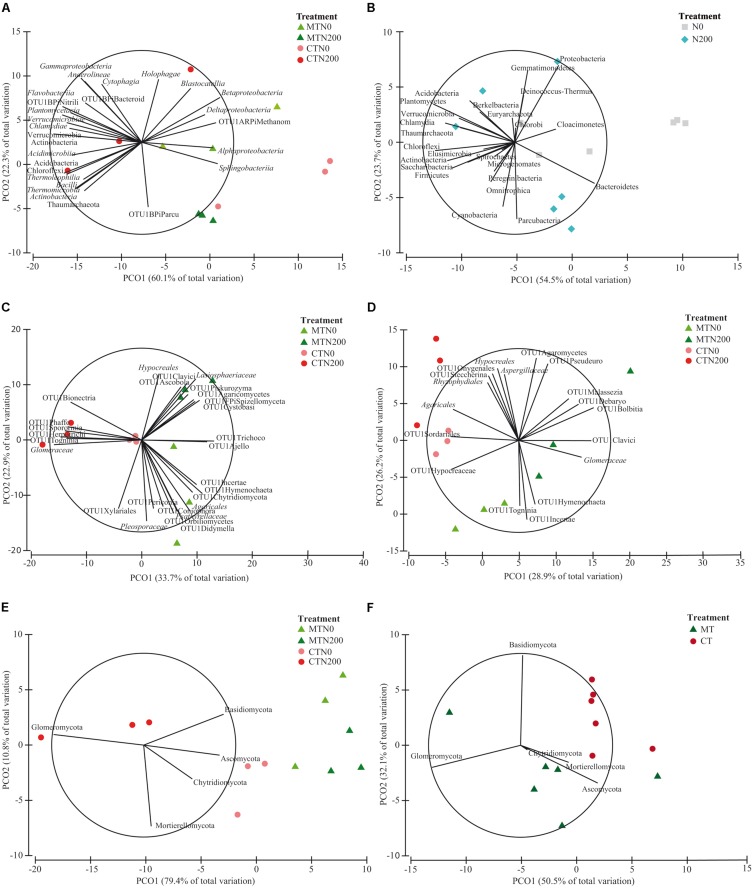
Principal Coordinates Analysis (PCO) biplots on the long-term effect of tillage and nitrogen fertilization on community diversity of prokaryotic classes at 0–15 cm soil depth **(A)**, prokaryotic phyla at 0–15 cm **(B)**, fungal families at 0–15 cm **(C)**, fungal families at 15–30 cm **(D)**, fungal phyla at 0–15 cm **(E)**, fungal families at 15–30 cm **(F)**. The output of the subfigures is based on the significant effect of treatments following the permutational analysis of variance. In each PCO biplot, we displayed only the phyla, classes and families with a strong correlation (*r* ≥ 0.70) with the ordination scores on each PCO axis. Data standardized to the median were square-root transformed and Bray-Curtis coefficients of similarity were calculated between samples.

The relative abundance of the other taxa at both soil depths was largely different among treatments, as shown in the pie/bar charts of Figures 1A, 2A. PERMANOVA showed that at 0–15 cm prokaryotic community structure at class level was significantly affected by N fertilization and by the interaction between tillage and N fertilization (Supplementary Table S4). In the PCO biplot, the first two principal coordinates explained 82.4% of the total variance (Figure 3A), and the variation partitioning analysis highlighted that N fertilization explained 22% of total variance (Table 1), in agreement with the fact that samples clearly clustered in the biplot in two groups along the first axis. However, while the community structure of soil prokaryotes under MTN0, MTN200 and CTN0 were almost similar (e.g., *Alphaproteobacteria*, *Sphingobacteriia*, *Betaproteobacteria*, *Deltaproteobacteria*), CTN200 showed a different prokaryotic community structure composed by many taxa (e.g., Chloroflexi, Verrucomicrobia, Actinobacteria, *Acidimicrobiia*, *Bacilli*, *Thermoleophilia*, *Thermomicrobia*, Thaumarchaeota) (Figure 3A). This was supported by the large amount of total variance explained by the interaction between tillage and N fertilization (50%) (Table 1). PERMANOVA showed that, at the same soil depth, prokaryotic community structure at phylum level was significantly affected only by N fertilization, which explained 24% of total variance (Table 1 and Supplementary Table S4), according to the total variance explained by the first principal coordinate (78.2% of the total variance) and to the sample clustering along the first axis (N0: Cloacimonetes; N200: Acidobacteria, Actinobacteria, Berkelbacteria, Chloroflexi, Plantomycetes, Thaumarchaeota, Verrucomicrobia etc.) (Figure 3B). Moreover, the output of PERMDISP for both analyses confirmed the differences in community dispersion between N fertilization treatments (Supplementary Table S4). By contrast, at 15–30 cm, no effect of tillage and N fertilization was reported on prokaryotic community structure at both class and phylum level.

**TABLE 1:** Variation partitioning of the long-term effect of tillage and nitrogen fertilization on soil prokaryotic and fungal diversity at different phylogenetic resolution and two soil depths, in a wheat-soybean rotation in the Mediterranean area.

	**0–15 cm**	**15–30 cm**
	**Variance explained**	**Variance explained**
*Prokaryotes at class level*		
TIL^a^	1.96^b^	–
N fert	**22.08**	–
TIL × N fert	**50.15**	–
*Prokaryotes at phylum level*		
TIL	8.38	–
N fert	**24.19**	–
TIL × N fert	29.72	–
*Fungi at family level*	
TIL	**25.26**	**24.20**
N fert	**18.36**	**14.81**
TIL × N fert	**22.89**	**27.14**
*Fungi at phylum level*		
TIL	**46.76**	**32.67**
N fert	**6.94**	−7.69
TIL × N fert	**33.88**	−8.02

*^*a*^Permutational analyses of variance were performed following a split-plot design with tillage (TIL) as main-plot factor and nitrogen fertilization (N fert) as subplot factor and with three replicate plots per treatment: TIL (minimum tillage and conventional tillage) and N fert (0 kg N ha^–1^ and 200 kg N ha^–1^). ^*b*^In bold statistically significant values (*P* ≤ 0.05).*

### Long-Term Effect of Conservation Tillage and N Fertilization on Community Diversity, Composition and Structure of Soil Fungi (Hypothesis 1)

As regard soil fungi, a lower variability due to treatments was observed respect to prokaryotes (Supplementary Table S3). In MT, N fertilization increased fungal richness at family level at 0–15 cm, whereas in CT it determined a decrease. Moreover, at the same phylogenetic resolution and soil depth, N fertilization did not modify the diversity indexes (*H’* and λ) under MT, whereas it decreased both indexes under CT. At 15–30 cm, tillage affected only λ at family level (MT < CT).

The total numbers of fungal families at 0–15 cm was 60, 67, 63, and 60 under MTN0, MTN200, CTN0 and CTN200, respectively, while at 15–30 was 64, 63, 63, and 62, respectively (Supplementary Figures S1G,H). At a higher phylogenetic resolution level, the number of fungal phyla at 0–15 and 15–30 cm was five for all treatments (Figure 2B). The relative abundance of the five phyla was as follows: Ascomycota (61%), Basidiomycota (18%), Glomeromycota (15%), Mortierellomycota (5%), and Chytridiomycota (1%). Looking at a higher phylogenetic resolution, the increasingly predominant classes of Ascomycota were *Leotiomycetes* (6%), *Eurotiomycetes* (6%), *Dothideomycetes* (24%), *Sordariomycetes* (62%), while those of the Basiodiomycota were *Exobasidiomycetes* (2%), *Microbotryomycetes* (2%), *Tremellomycetes* (12%) and *Agaricomycetes* (82%) (Figures 2C,D). By contrast, each of the other classes accounted for less than 1%. Although the majority of fungal taxa were common to all treatments at both soil depths (0–15 cm: 53 families; 15–30 cm: 59 families), some were exclusively found in each treatment (Supplementary Figures S1G,H). Specifically, at 0–15 cm, the families *Coniophoraceae* (#1; Basidiomycota) and *Debaryomycetaceae* (#26; Ascomycota) were retrieved in MTN0 and in MTN200, respectively. At 15–30 cm, the families *Geastraceae* (#7; Basidiomycota), *Cordycipitaceae* (#19; Ascomycota) and *Steccherinaceae* (#9; Basidiomycota) were exclusively retrieved in MTN0, CTN0 and CTN200, respectively. However, these unique taxa occurred at very low relative abundances (data not shown).

The relative abundance of the other taxa at both soil depths was largely different among treatments as shown in the pie/bar charts of Figures 1B,C, 2B–D. PERMANOVA showed that fungal community structure at family and phylum level was significantly affected by tillage, N fertilization and the interaction between tillage and N fertilization at 0–15 cm (Supplementary Table S4). Looking at corresponding PCO biplots, the first two principal coordinates explained 56.6 and 90.2% of the total variance, respectively (Figures 3C,E). At family level, tillage, N fertilization and their interaction explained 25, 18 and 23% of total variance, respectively, while at phylum level, the same treatments explained 47, 7, 34% of total variance, respectively (Table 1). At family level, samples belonging to MT and CT clearly clustered into two separated groups along the first axis (PCO1: 33.7% of total variance) (Figure 3C). Within MT, samples belonging to N0 and N200 were separated in two clusters along the second axis (PCO2: 22.9% of total variance), showing a strong difference of their fungal community structure (e.g., MTN0: OTU1Chytridiomycota, OTU1Coniophora, *Pleosporaceae*, OTU1Didymella; MTN200: *Lasiophaeriaceae*). Within CT, many fungal families were commonly found in N0 and N200, but their relative abundances were consistently higher in N200 than in N0 (e.g., *Glomeraceae*, OTU1Herpotrichi and OTU1Phaffo). At phylum level, samples belonging to MT and CT clearly clustered into two separated groups along the first axis (PCO1: 79.4% of total variance), while samples belonging to N0 and N200 clustered into two separated groups along the second axis (PCO2: 10.8% of total variance) (Figure 3E). Basidiomycota, Ascomycota and Chytridiomycota were highly abundant in MTN0, MTN200 and CTN0, whereas Glomeromycota in CTN200.

PERMANOVA showed that fungal community structure at family level was significantly affected by tillage, N fertilization and the interaction between tillage and N fertilization at 15–30 cm, whereas at phylum level only by tillage (Supplementary Table S4). At family level, tillage, N fertilization and their interaction explained 24, 15, 27% of total variance, respectively, whereas at phylum level tillage explained 33% of total variance (Table 1). At family level, MT and CT samples clustered into two separated groups along the first axis (PCO1: 28.9% of total variance) (Figure 3D). Within MT, samples under N0 and N200 were separated in two clusters along the second axis (PCO2: 26.2% of total variance) (e.g., MTN0: OTU1Hymenochaeta, OTU1 Togninia; MTN200: *Glomeraceae*, OTU1Malassezia and OTU1Debaryo). Within CT, samples under N0 and N200 were more similar than in MT, although in N0 some fungal families were more abundant than in N200 (e.g., *Agaricales*, OTU1Sordariales, OTU1Steccherina). At phylum level, MT and CT samples clustered into two groups along the first axis (PCO1: 50.5% of total variance), with Glomeromycota highly abundant in MT (Figure 3F). PERMDISP for all the significant analyses generally confirmed the differences in fungal community dispersion (Supplementary Table S4).

### Role of Soil Depth in Shaping Diversity, Composition and Structure of Soil Prokaryotes and Fungi (Hypothesis 2)

Richness, *H’* and λ of prokaryotes at both class and phylum level were higher at 0–15 than at 15–30 cm (Supplementary Table S5). As regard fungi, no differences were observed according to soil depth at both family and phylum levels.

The majority of prokaryotic and fungal taxa were common to both soil depths (prokaryotes: 51 classes and 22 phyla; fungi: 66 families) (Supplementary Figures S1C,F,I). The prokaryotic taxa exclusively found at 0–15 cm and 15–30 cm were the phylum Berkelbacteria (#47) and the class *Methanobacteria* (#52; Euryarchaeota), respectively (Figure 1A). The fungal taxa exclusively found at 0–15 cm were the families *Coriolaceae* (#10; Basidiomycota) and *Debarymycetaceae* (#26; Ascomycota). These unique taxa occurred at very low relative abundances (data not shown).

**FIGURE 4:**
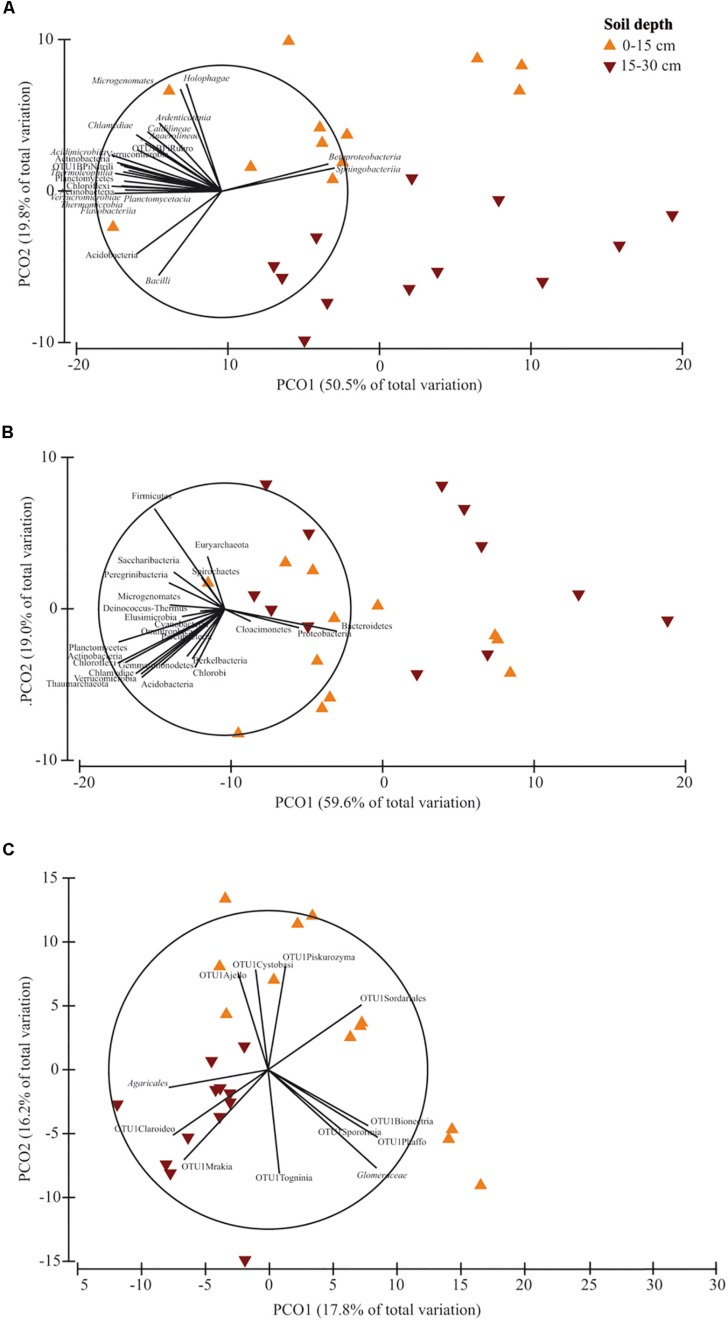
Principal Coordinates Analysis (PCO) biplots on the effect of soil depth (0–15 *vs.* 15–30 cm) on community diversity of prokaryotic classes **(A)**, prokaryotic phyla **(B)** and fungal families **(C)**. Tillage and nitrogen fertilization were used as covariates. The output of the subfigures is based on the significant effect of soil depth following the permutational analysis of variance. In each PCO biplot, we displayed only the phyla, classes and families with a strong correlation (*r* ≥ 0.70) with the ordination scores on each PCO axis. Data standardized to the median were square-root transformed and Bray-Curtis coefficients of similarity were calculated between samples.

PERMANOVA showed that soil depth had a significant effect on prokaryotic community structure at class and phylum level, whereas it affected fungal structure only at family level (Supplementary Table S6). In the corresponding PCO biplots, the first two principal coordinates explained 70.3, 78.6 and 34.0% of the total variance (Figure 4), and the variation partitioning analysis highlighted that soil tillage depth explained 37, 10, 21% of total variance, respectively (Table 2), in agreement with the fact that samples clearly and consistently clustered in the biplots in two groups along the second axis. For prokaryotes, the biplots highlighted that almost all the families and phyla were more abundant at 0–15 cm than at 15–30 cm (Figures 4A,B). For fungi, the community structure at 0–15 cm was characterized by *Glomeraceae* (Glomeromycota), OTU1Sordariales (Ascomycota), OTU1 Phaffo (Ascomycota), whereas the community structure at 15–30 cm was characterized by *Agaricales* (Basidiomycota), and OTU1Mrakia (Basidiomycota). PERMDISP for all the analyses confirmed the differences in community dispersion between the two soil depths (Supplementary Table S6).

**TABLE 2:** Variation partitioning of the effect of soil depth (0–15 *vs.* 15–30 cm) on soil prokaryotic and fungal diversity at different phylogenetic resolution in a wheat-soybean rotation in the Mediterranean area.

	**0–15 *vs.* 15–30 cm**
	**Variance explained**
*Prokaryotes at class level*	
Soil depth ^a^	**36.87** ^b^
TIL	5.90
N fert	**7.14**
*Prokaryotes at phylum level*
Soil depth	**10.32**
TIL	**5.10**
N fert	**5.78**
*Fungi at family level*		
Soil depth	**21.21**
TIL	**7.47**
N fert	**7.19**
*Fungi at phylum level*		
Soil depth	–
TIL	–
N fert	–

*Tillage and nitrogen fertilization were used as covariates. ^*a*^Permutational analyses of variance were performed using soil depth as fixed factor, tillage (TIL) and nitrogen fertilization (N fert) as covariates and 12 replicates plots per soil depth. TIL, minimum tillage and conventional tillage; N fert: 0 kg N ha^–1^ and 200 kg N ha^–1^. ^*b*^In bold statistically significant values (*P* ≤ 0.05).*

### Linking Soil/Yield Parameters to Microbial Community Structure (Hypothesis 3)

**FIGURE 5:**
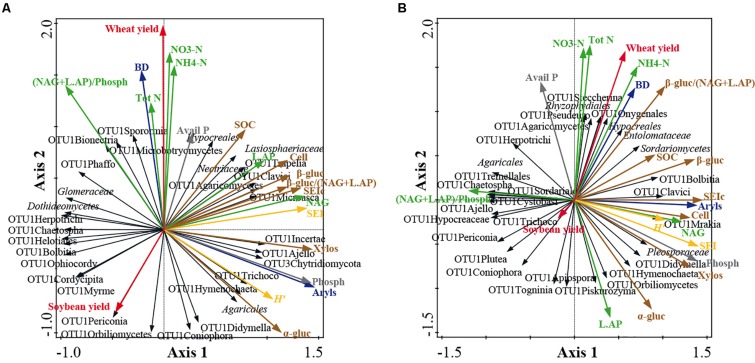
Co-inertia biplots on fungal community structure at family level and soil physico-chemical and enzymatic parameters at 0–15 cm soil depth **(A)** and at 15–30 cm soil depth **(B)**. See Figures 1B,C and Supplementary Table S2 for fungal taxon assignment. The colored arrows represent the soil parameters: bulk density (BD) and arylsulphatase (Aryls) (blue arrows); wheat and soybean yields (red arrows); P cycle parameters: available P, Avail P; phosphatase, Phosph (gray arrows); N cycle parameters: total N, Tot N; ammonium, NH_4_-N; nitrate, NO_3_-N; chitinase, NAG; Leucine, AP, L.AP; ecoenzymatic N/P acquisition activity, (NAG + L.AP)/Phosph (green arrows); C cycle parameters: soil organic carbon, SOC; cellulose, Cell; α-glucosidase, α-gluc; β-glucosidase, β-gluc; xylosidase, Xylos; synthetic enzyme index of C cycle, SEIc; ecoenzymatic C/N acquisition activity, β-gluc/(NAG + L.AP) (brown arrows); enzyme indexes: synthetic enzyme index, SEI; Shannon index, *H*’ (yellow arrows). The black arrows represent fungal taxa. The lengths of the vector arrows indicate the influence of the parameters on the co-inertia co-structure.

Co-inertia analyses highlighted significant relationships between soil/yield parameters and fungal community structure at family level at both soil depths (Figure 5). Co-inertia values were 0.253 and 0.209 at 0–15 and 15–30 cm, respectively. The significance of the relationships between data sets was verified by the Mantel test (0–15 cm: *r* = 0.314, *P* = 0.031; 15–30 cm: *r* = 0.304; *P* = 0.050). At 0–15 cm soil depth, the axis 1 and axis 2 loadings of the first step account for 25.9% and 18.6% of the total variance, respectively (Figure 5A) and at 15–30 cm for 22.2 and 14.4% of the total variance, respectively (Figure 5B). Moreover, at 0–15 cm, the axis 1 and axis 2 loadings of the second step account for 57.0 and 28.8% of the total variance, respectively (Figure 5A) and at 15–30 cm for 57.7 and 27.6% of the total variance, respectively (Figure 5B).

At both soil depths, the C parameters discriminated the fungal community structure along axis 1, whereas N parameters and crop yields mostly discriminated along axis 2 (Figure 5). Moreover, at both soil depths, all C-cycling enzymes were positively related to SOC. At 0–15 cm, the fungal taxa positively associated to C parameters were: *Lasiospheraceae*, OTU1Clavici, OTU1Agaricomycetes, OTU1Microasca and OTU1Hymenochaeta (Figure 5A). At 15–30 cm, the fungal taxa positively associated to C parameters were: *Sordariomycetes*, *Pleosporaceae*, OTU1Bolbitia, OTU1Clavici, OTU1Didymella and OTU1Hymenochaeta (Figure 5B).

At 0–15 cm, all N enzymes were positively related to N in soil (N tot, NO_3_-N and NH_4_-N), whereas at 15–30 cm they were negatively related (Figure 5). At 0–15 cm, the fungal taxa positively associated to N parameters were: *Nectriaceae*, OTU1Trapelia, OTU1Sporormia, and OTU1Microbotryomycetes (Figure 5A). At 15–30 cm, the fungal taxa associated to N parameters were: OTU1Steccherina, *Rhyzophydiales* and OTU1Agiospora (Figure 5B). At 0–15 cm soil depth, N in soil was positively related to wheat yield and negatively with soybean yield, whereas at 15–30 cm N in soil was positively related only to wheat yield (Figure 5).

Several taxa were negatively related to C and P parameters at 0–15 cm (e.g., *Glomeraceae, Dothideomycetes*, OTU1Herpotrichi and OTU1Bolbitia) and at 15–30 cm (e.g., OTU1Periconia, OTU1Hypocreaceae, OTU1Ajello and *Agaricales*) (Figure 5). Moreover, some other taxa were negatively related to N in soil at 0–15 cm (e.g., OTU1Coniophora, OTU1Orbiliomycetes and OTU1Didymella) (Figure 5A). By contrast, no significant relationships were observed along the soil profile for prokaryotes at both phylogenetic resolutions and for fungi at phylum level (data not shown).

## Discussion

Here, using the high-throughput resolution of Illumina sequencing, we found that (i) the majority of prokaryotic and fungal taxa were common to all treatments at both soil depths; (ii) few bacterial taxa (Cloacimonates, *Spirochaetia* and Berkelbacteria) and a larger number of fungal taxa (i.e., *Coniphoraceae*, *Debaryomycetaceae*, *Geastraceae*, *Cordicypitaceae* and *Steccherinaceae*) were unique to specific management practices; (iii) soil prokaryotic and fungal structure was heavily influenced by the interaction of tillage and N fertilization; (iv) the prokaryotic community structure of the fertilized conventional tillage system was remarkably different respect to the unfertilized conservation and conventional systems in the surface layer; (v) the effect of N fertilization in shaping the fungal community structure of the surface layer was higher under conservation tillage systems than under conventional tillage systems; (vi) soil microbial community was shaped by soil depth irrespective of the effect of plowing and N addition; (vii) chemical and enzymatic parameters of soil and crop yields were significantly related to fungal community structure along the soil profile.

### Long-Term Effect of Conservation Tillage and N Fertilization on Diversity of Soil Prokaryotes and Fungi

The diversity of soil prokaryotes at class and phylum level, as measured by Shannon and Simpson index, at 0–15 cm soil depth was increased by N fertilization (+2%). It is possible to infer that in the surface layer, where tillage disturbs soil, N fertilization increases the homogeneity among taxa as well as the occurrence of the most common taxa. Accordingly, de Graaff et al. (2019) found in several field studies across the world, including the Mediterranean area, that N fertilization increased soil bacterial diversity by 3%. This result was explained by the addition of N organic fertilizers, alone or in combination with mineral fertilizers. It is interesting to highlight that, in line with the length of our experiment, the duration of N mineral fertilizer addition (≥5 years) was pointed out as a major determinant for the increase of soil bacterial diversity.

By contrast, N fertilizer addition decreased soil fungal diversity up to 5% (richness, *H’* and λ) in the surface layer under the plowed system (CT) and not in the disk-harrowed system (MT), where only fungal richness was promoted (+7%). To date, the effects of N enrichment on fungal diversity was less studied in agricultural soil compared to forests (e.g., Wallenda and Kottke, 1998; Eom et al., 1999; Egerton-Warburton and Allen, 2000; Peter et al., 2001; Avis et al., 2003; Frey et al., 2004; Jumpponen and Johnson, 2005; Antoninka et al., 2011; Lin et al., 2012). Many of those studies were based on the assessment of ecto- and endo-mycorrhizal diversity and not on total fungi. Recently, Leff et al. (2015), by analyzing the soil fungal diversity of 25 globally distributed grasslands, observed that Shannon index responds weakly to N addition, although the diversity of Glomeromycota was consistently reduced. In our study, the reduction of total fungal diversity may be explained by a toxic effect of urea due to its hydrolyzation after application, leading to a net decrease of soil pH (Omar and Ismail, 1999). In addition, the different response of fungal diversity indexes (*H’* and λ) to N fertilization between minimum and conventional tillage may be driven by the increase of fungal richness observed under N fertilization in MT respect to the reduction in CT.

In the subsurface layer (15–30 cm soil depth), the richness and *H’* of prokaryotes at phylum level was increased in CT compared to MT, supporting the idea that plowing is not detrimental for the diversity of soil prokaryotes. The higher BD found in deeper soil layers in MT systems results in a reduction of pore size and soil aeration, causing local anoxic conditions and therefore the reduction of the diversity of prokaryotes (Berisso et al., 2012).

### Long-Term Effect of Conservation Tillage and N Fertilization on Community Composition of Soil Prokaryotes and Fungi

A conserved core community of soil prokaryotic and fungal taxa was retrieved across treatments at both soil depths and at different phylogenetic resolutions. Indeed, averaged over soil depths, 85% and 81% of total prokaryotes at class and phylum resolution, respectively, were shared among treatments, whereas 85% of total fungi were shared at family level. The fact that soil microbes are strongly conserved throughout these long-term treatments is surprising and noticeable.

Interestingly, at 0–15 cm soil depth, the bacterial phylum Cloacimonetes and the class *Spirochaetia* were the taxa uniquely found in unfertilized conventional tillage (CTN0), whereas the newly recognized phylum Berkelbacteria (Wrighton et al., 2014) was uniquely found in conventional fertilized tillage (CTN200). Cloacimonetes is a mesophylic phylum able to degrade cellulose and produce methane, while the members of *Spirochaetia* degrade cellulose and chitin (Limam et al., 2014; Chojnacka et al., 2015; Dai et al., 2016; Deng et al., 2018) and anaerobically decompose organic matter (Baba et al., 2014). Moreover, the phylum Berkelbacteria is an ATP binding and a putative transcriptional regulator with helix-turn-helix (HTH)-like protein. By contrast, at 15–30 cm soil depth, the phylum Cloacimonetes and the class *Spirochaetia* were the taxa uniquely found in fertilized minimum tillage (MTN200). These results point at the overall metabolic flexibility of these groups of bacteria or at the presence of different members belonging to the same group, but possessing diverse metabolic potential.

As regard fungi, the Basidiomycota family *Coniophoraceae*, exclusively found under MTN0 in the surface layer, includes saprophytic brown-rot fungi (Thorn et al., 1996; Lee et al., 2004; Utobo and Tewari, 2015), as well as the mycorrhizal fungi *Boletales* spp., and other members known to be cellulolytic. Moreover, the Ascomycota family *Debaryomycetaceae*, exclusively found under MTN200 in the surface layer, includes xylose-fermenting yeasts (Steindorff et al., 2016) *Cordycipitaceae*, exclusively found in the subsurface layer of CTN0 and CTN200, is a family belonging to Ascomycota including entomophatogenic fungi (Vega et al., 2009), whereas the *Steccherinaceae*, found in the same conditions, is a family belonging to Basidiomycota, includes fungi that produce proteinase (Kudryavtseva et al., 2008). Finally, *Geastraceae*, exclusively found in MTN0, are a family belonging to Basidiomycota and including fungi that decay SOM and recycle C and N, also acting as biofertilizers (Eriksson et al., 1990; Gadd, 2001).

### Long-Term Effect of Conservation Tillage and N Fertilization on Community Structure of Soil Prokaryotes and Fungi

In recent years, using molecular tools, such as Sanger or next-generation sequencing, the effect of tillage or N fertilization were explored to give indications on management practices that support beneficial taxa and suppress detrimental ones (Hartmann et al., 2015; Francioli et al., 2016). These studies assessed soil bacteria and fungi (e.g., tillage and bacteria/fungi: Mirás-Avalos et al., 2011; Bevivino et al., 2014; Sharma-Poudyal et al., 2017; N fertilization and bacteria/fungi: Lienhard et al., 2014; Ding et al., 2017; Li et al., 2017; Wang et al., 2018). Other studies focused on the interactions of tillage or N fertilization with crop rotation (Degrune et al., 2017; Ai et al., 2018). Only one study, carried out in a long-term vineyard experiment in Germany, found the interaction between soil tillage and N fertilization as major explanatory variable for the shift of soil bacterial and fungal communities (Pingel et al., 2019). In our study, despite the high degree of similarity in term of microbial composition among treatments, we firstly found that the prokaryotic and fungal community structure is strongly shaped by the interaction of tillage and N fertilization. However, N fertilization played a crucial role in shaping prokaryotic and fungal assemblages, whereas for soil fungi tillage was the additional main driver. In our study, *Alpha*-, *Beta*-, and *Delta-proteobacteria* showed higher relative abundances at the surface layer in the minimum soil-disturbed system irrespective of N fertilization as well as in the plowed unfertilized one. *Alphaproteobacteria* is a class important for cellulose hydrolyzation, not affected by the addition of N/P and known to actively fix N_2_ (e.g., Rhizobia) (Pankratov et al., 2006). As regard the fertilized conventional tillage, we found a high number of abundant taxa compared to the other treatments, such as *Acidimicrobiia*, Actinobacteria, *Bacilli*, Chloroflexi, *Gammaproteobacteria* and Verrucomicrobia. Among these taxa, the high abundance of the class *Acidimicrobiia* and of the whole phylum Acidobacteria may have been determined by a reduction of pH with urea application (Omar and Ismail, 1999). Acidobacteria were described as low-growing oligotrophics (or K-selected) and less present with high concentration of labile C (Fierer et al., 2007), and were found highly present at low N fertilization rates in several studies (Fierer et al., 2007; Ramirez et al., 2010; Eo and Park, 2016; Francioli et al., 2016; Wang et al., 2018). This inconsistency can be explained by the fact that not all the members of the phylum can be considered distinctly copiotrophic or oligrotrophic and therefore their functional behavior can be variable. The members of the phylum Actinobacteria are well-known decomposers, chitin utilizers, rhizosphere colonizers and strongly responding to root exudates (DeAngelis et al., 2008, 2009). Similar to results of our study, Actinobacteria were shown to increase with high N input (Ramirez et al., 2010). The high presence of *Bacilli* is positive since they cohabit in the rhizosphere with AMF and promote plant growth by producing phytohormones (e.g., auxins and gibberellins) (Gutiérrez-Manero et al., 1996; Gutiérrez-Mañero et al., 2001; Medina et al., 2003; Ramesh et al., 2014). In agreement with our results, Chlroflexi were found abundant under fertilized systems (Francioli et al., 2016; Jiménez-Bueno et al., 2016). This phylum includes filamentous bacteria playing a role in anaerobic ammonium oxidation, degradation of SOM, using sucrose, glucose and N-acetyl-glucosamine (Kragelund et al., 2007; Kindaichi et al., 2012). *Gammaproteobacteria* is a class generally found under high N level following manure application, playing a crucial role in the decomposition of labile C compounds and in the control of bacterial pathogens (Fierer et al., 2007; Eo and Park, 2016; Francioli et al., 2016). Finally, the phylum *Verrucomicrobia* was found to be influenced by soil management and nutritional regimes (Buckley and Schmidt, 2001; Kielak et al., 2009).

By looking at the prokaryotic shifts at a lower level of resolution (phylum level), we could detect only the effect N fertilization, supporting the fact that it is important to utilize molecular tools at high resolution to allow a deeper discrimination.

As regards soil fungi, we found a strong interaction between tillage and N fertilization not only in the surface layer, as for prokaryotes, but also in the subsurface layer, confirming that this eukaryotic community could be more sensitive to management practices respect to prokaryotes (Degrune et al., 2017; Sharma-Poudyal et al., 2017). At the surface layer, the phylum Chytridiomycota and the families *Coniophoraceae* and *Pleosporaceae* were highly abundant in MTN0. Chytridiomycota comprises either saprophytic or parasitic fungi that degrade cellulose and secrete many extracellular enzymes (Gulis et al., 2008; Kameshwar and Qin, 2016). In line with our results, the family *Pleosporaceae*, belonging to the class of *Dothideomycetes*, which comprises many saprophytic and plant pathogens (e.g., *Alternaria* spp.), was not affected or decreased by mineral fertilization (Freedman et al., 2015; Francioli et al., 2016; Ding et al., 2017). On the other hand, we found the family *Lasiosphaeraceae*, which includes wood-decaying fungi (Nordeìn and Paltto, 2001; Cannon and Kirk, 2007), highly abundant under MTN200, and this is in line with previous results (Hartmann et al., 2015; Ding et al., 2017). Moreover, N fertilization under conventional tillage increased the abundance of the Ascomycota families *Phaffomycetaceae*, including yeasts, utilizing different N sources (Linder, 2019), and *Herpotrichiellaceae* highly abundant under N fertilized conditions (Weber et al., 2013; Ding et al., 2017). Although it was expected that reduced soil disturbance and low N availability would be more beneficial for AMF (Liu et al., 2015; Mbuthia et al., 2015; Bowles et al., 2017), we surprisingly found a strong effect of the interaction between tillage and N fertilization. Indeed, *Glomeraceae* at the surface layer were more abundant under CTN200 compared to the other treatments and at the subsurface layer they were more abundant under MTN200. The positive effect of N fertilization on *Glomeraceae* is likely due not to the direct effect of increase of N availability, but to the indirect effect of promotion of plant growth and root size. In the surface layers, where the majority of roots are concentrated, loosed and aerated soil promote highly competitive taxa able to rapidly recover following tillage, while in the subsurface hard soil, prevail taxa able to tolerate low oxygen availability, but highly sensitive to the disturbance caused by tillage (Jansa et al., 2002; Avio et al., 2006; Voets et al., 2006).

### Role of Soil Depth in Shaping Diversity, Composition and Structure of Soil Prokaryotes and Fungi

Although it is widely acknowledged that soil microbes affect nutrient cycling along the soil profile, to date the understanding of the diversity, composition and structure of soil prokaryotic and fungal communities is mainly limited to the surface layer, with the majority of studies targeting the up 15 cm. The higher diversity of prokaryotes found in this study in the surface layer respect to the deeper layer was previously reported by Eilers et al. (2012). By contrast, the similarity in the fungal diversity we found along the soil profile is in disagreement with previous studies that reported a declining trend in the whole fungal and AMF diversity with increasing soil depth (Oehl et al., 2005; Jumpponen et al., 2010).

Moreover, the fact that the fungal family *Coriolaceae* was exclusively found at 0–15 cm is positive since this group of wood-decaying white-rot fungi are able to secrete hydrolytic (e.g., endoglucanase and endoxylanase) and oxidative (e.g., manganese peroxidase and laccase) extracellular enzymes (Hofrichter, 2002; Levin et al., 2007). In addition, the prokaryotic class *Methanobacteria*, belonging to the largely less explored Archaea phylum Euryarchaeota, was uniquely found at 15–30 cm, in agreement with their role to reduce nitrate (Cabello et al., 2004).

Looking at prokaryotic and fungal structure at class/family resolution, we found strong differences between the two depths, irrespective of tillage and N fertilization. This is in agreement with previous studies (Blume et al., 2002; Griffiths et al., 2003; Hartmann et al., 2009). Root morphology and therefore exudation deeply vary along the soil profile (Wen et al., 2019) and this can be considered a major factor shaping microbial structure. Another factor can be the indirect effect of AMF in soil and within roots along the soil profile (Kabir et al., 1998; Oehl et al., 2005; Jones et al., 2018). This is also supported by our data showing a high relative abundance of *Glomeraceae* at 0–15 cm and of *Claroideoglomeraceae* at 15–30 cm.

### Linking Soil/Yield Parameters to Microbial Community Structure

Several attempts were done in order to associate soil microbial community structures and soil parameters and/or plant species (i.e., Johnson et al., 2004; Berg and Smalla, 2009; Zhu et al., 2012; Ciccolini et al., 2015, 2016b). However, these attempts were based on the implicit assumption that microorganisms are unilaterally affected by soil parameters, crop residues or species identity/communities. Actually, microorganisms are also the main players in nutrient cycling, determining nutrient accumulation, and soil structure (Li et al., 2017; Sahu et al., 2017; Powell and Rillig, 2018). Indeed, the co-inertia analyses we performed aimed to disentangling the roles of microbial taxa in soil nutrient cycling and crop yield and to clarify how these roles vary accordingly to soil conditions. The analyses highlighted meaningful bilateral interactions with soil parameters and crop yields only for soil fungi at low taxonomic level (i.e., family level). At higher taxonomic resolution (i.e., order, class, phylum), it was not possible to detect the fungal/bacterial taxa playing functional roles in nutrient cycling and yield production. This is due to the heterogeneous composition of taxa at high taxonomic level, in terms of roles and ecosystem services (Fra̧c et al., 2018).

At surface layer, *Lasiospheraceae*, the fungal family belonging to Ascomycota that we found positively associated to C-cycling parameters, was previously described as saprobes only in soil of temperate forests (Cannon and Kirk, 2007). Other Ascomycota, positively related to C, were the families *Clavicipitaceae* and *Microascaceae* that were previously described as biotrophics/necrotrophs of other fungi and having a high affinity for materials of animal origin (Mycology Web pages of the New Brunswick Museum, 2019). Moreover, also members of the phylum Basidiomycota, such the class *Agaricomycetes* and the family *Hymenochaetaceae*, were highly and positively associated to SOC and enzymes linked to C-cycling. The members of *Agaricomycetes* are symbionts (ectomycorrhizal fungi) and saprobes playing a key role in lignocellulose decomposition (Kersten and Cullen, 2013), while the members of *Hymenochaetaceae* are described as parasites or saprobes (Mycology Web pages of the New Brunswick Museum, 2019). By contrast, at the subsurface layer, *Pleosporaceae* and *Didymellaceae* (phylum Ascomycota), previously described as saprobes as well as plant pathogens (Rodriguez et al., 2009), were the fungal families positively associated to the C cycle. In such a layer, main players of the C-cycling were also members of the family *Bolbitiaceae* (phylum Basidiomycota) that were already described as saprobes and symbionts (ectomycorrhizal fungi) (Cannon and Kirk, 2007). Moreover, the occurrence of members of the families *Clavicipitaceae* and *Hymenochaetaceae* also in the subsurface layer supports their key role in C-cycling along the profile.

At surface layer, the families *Nectriaceae* and *Trapeliaceae*, belonging to the phylum Ascomycota, and previously shown to play important roles in organic matter decomposition and being associated with green algae, respectively (Zhou et al., 2017), were positively related to N-cycling parameters, together with the ascribed saprobes *Sporormiaceae* (phylum Ascomycota, *Pleosporales*) and *Microbotryomycetes* (phylum Basidiomycota) (Cannon and Kirk, 2007). By contrast, at the subsurface layer, the main players in N cycle were members of the family *Steccherinaceae* (phylum Basiomycota) that were previously described as soil saprobes producing protolithic enzyme, such as protease (Kudryavtseva et al., 2008), and members of the order *Rhizophydiales* (phylum Chytridiomycota), parasites of plants, algae, protists and invertebrates. Therefore, our results support the fact that different fungal taxa play key roles in N and C cycles along the soil profile. This can be well explained taking into consideration the soil physical conditions, such as porosity, moisture, temperature and air diffusion that provide different microhabitats for soil microbes (Horn et al., 1994). The differential production of exudates from the root system mainly concentrated in the surface layer may therefore modify the patterns of accumulation of SOM and the concentration of ammonium and nitrate. Our results confirm previous conspicuous data reporting that AMF biomass in roots and soil is markedly decreased by high P availability and C rich substrates (Abbott et al., 1984; Smith et al., 2011). In addition, the quality of residues (wheat and soybean), highly discriminating the fungal structures at both soil depths, may be strongly related to the quality of crop residues, as previously found (Pellegrino et al., 2011, 2015a).

## Conclusion

This study circumvented the problem of the highly complexity of microbial diversity in soil for evaluating the influence of agricultural management by an appropriate plot size, homogenizing soil samples and using a long-term field trial. A conserved core of prokaryotic and fungal taxa across tillage and N fertilizer treatments was identified. However, the interaction between tillage systems and N fertilizion strongly shifted soil prokaryotic and fungal community structure toward a higher presence of functional taxa linked to nutrient cycling, with positive implications on wheat yield. Indeed, the increase of wheat yield was associated to the soil parameters linked to N cycle and to specific fungal taxa (e.g., *Nectriaceae*, *Trapeliaceae*, *Steccherinaceae*), whereas the increase of SOC was associated to the other fungal taxa (e.g., C cycle: *Lasiospheraceae, Microascacea*, *Plesoporaceae*). The families *Clavicipitaceae* and *Hymenochaetaceae* were consistently associated to C cycle along the soil profile. These results, only detectable for fungi at low taxonomic level, are important advances for supporting cropping systems based on minimum tillage and adequate N fertilization in the Mediterranean area. However, whether these results are of general validity in different soil types across the Mediterranean area is still a matter for further investigation based on the identification of taxa at a lower taxonomic resolution (i.e., species) or ‘omics approaches that allow to measure gene expression linked to nutrient cycling.

## Data Availability

The datasets generated for this study can be found in the NCBI Sequence Read (SRA) database (prokaryotic accession numbers MK903871–MK904485; fungal accession numbers MK881790–MK881896).

## Author Contributions

EP and GP contributed to designing the experiments, collecting the data, analyzing the data, and writing the manuscript. LE contributed to designing the experiments and discussing the data. MN contributed to discussing the data. All authors reviewed and approved the manuscript before its submission.

## Conflict of Interest Statement

The authors declare that the research was conducted in the absence of any commercial or financial relationships that could be construed as a potential conflict of interest.

## Footnotes

1 https://www.megasoftware.net

2 http://bioinfogp.cnb.csic.es/tools/venny/
