# Supplementary material for: Interaction Between Conservation Tillage and Nitrogen Fertilization Shapes Prokaryotic and Fungal Diversity at Different Soil Depths: Evidence From a 23-Year Field Experiment in the Mediterranean Area
Source: Front Microbiol. 2019 Sep 4;10:2047. doi: 10.3389/fmicb.2019.02047 (PMC6737287; doi:10.3389/fmicb.2019.02047)
Supplement: Supplementary file 1 [file Data_Sheet_1.docx]

| **TABLE S1 \|** Primer sets used for amplification of prokaryotes and fungi in soil and relative sequence, targeted genes, PCR reaction conditions and references. | | | | | | | | |
| --- | --- | --- | --- | --- | --- | --- | --- | --- |
| **Soil microbial community** | **Primer set ^a^** | | **Sequence (5'-3')** | **Targeted gene ^b^** | **PCR reaction conditions ^c^** | | | **References** |
| Prokaryotes | F | Bac515F-Y-ill | TCGTCGGCAGCGTCAGATGTGTATAAGAGACAGA NNNHNNNWNNNH GTGYCAGCMGCCGCGGTAA | V4 region 16 SSU rRNA gene | Initial denaturation: 95°C for 2 min | | | Modified from Apprill et al., 2015; Parada et al., 2016. |
|  |  |  |  |  | Denaturation: 94°C for 30 s |  | 30 cycles |  |
|  | R | Bac806rmod-ill | GTCTCGTGGGCTCGGAGATGTGTATAAGAGACAGGGACTACNVGGGTWTCTAAT |  | Annealing: 53°C for 45 s |  |  |  |
|  |  |  |  |  | Elongation: 72°C for 1 min and 30 s |  |  |  |
|  |  |  |  |  | Final elongation: 72°C for 10 min | | |  |
| Fungi | F | ITS1F | CTTGGTCATTTAGAGGAAGTAA | ITS1-5.8S-ITS2 region | Initial denaturation: 95°C for 2 min | | | White et al., 1990; Gardes and Bruns, 1993; Ihrmark et al., 2012. |
|  |  | gITS7-ill | TCGTCGGCAGCGTCAGATGTGTATAAGAGACAGA NNNHNNNWNNNH GTGARTCATCGARTCTTTG |  | Denaturation: 94°C for 30 s |  | 30 cycles |  |
|  | R |  |  |  | Annealing: 55°C for 45 s |  |  |  |
|  |  | ITS4 | TCCTCCGCTTATTGATATGC |  | Elongation: 72°C for 1 min and 30 s |  |  |  |
|  |  | ITS4-ill | GTCTCGTGGGCTCGGAGATGTGTATAAGAGACAGTCCTCCGCTTATTGATATGC |  | Final elongation:72°C for 10 min | | |  |
| ^a^F: forward primer; R: reverse primer; | | | | | | | | |
| ^b^SSU rRNA: small subunit ribosomal RNA; ITS: internal transcribed spacer; | | | | | | | | |
| ^c^For characterizing the communities of soil fungi, the amplification of the targeted gene was performed using a semi-nested PCR with ITS1F/ITS4 primer pair as the primary PCR. Thus, the ten-times diluted products of the first PCR reaction were used for the amplification by gITS7-ill/ITS4-ill primer pair. PCR reaction conditions were equal for both primer sets. | | | | | | | | |

**Supplementary Materials and methods**

**Soil physical, chemical and biochemical analyses**

Samples collected for soil bulk density (BD) were dried at 105°C until constant weight and BD was assessed following Blake and Hartge (1986). Soil samples sieved at 2 mm were analysed for soil organic carbon (SOC; CHN combustion method, LECO, Italy), total nitrogen (Total N; macro Kjeldahl digestion procedure, Bremner and Mulvaney, 1982) and available P (Avail P; colorimetry using a solution of sodium bicarbonate, Olsen et al., 1982). Ammonium (NH_4_-N) was assessed photometrically using the Spectroquant® kit (Merck Millipore Corp., Billerica, MA, USA) and nitrate (NO_3_-N) using the ultraviolet spectrophotometric technique (Goldman and Jacobs, 1961).

Soil enzyme potential activities were measured using the fluorogenic methylumbelliferyl (MUF)-substrates method (Marx et al., 2001; Vepsäläinen et al., 2001). The following hydrolytic enzymes, known to be involved in C, N, P and S biogeochemical cycles, were analyzed for β-cellobiohydrolase = cellulase (Cell; EC 3.2.1.91), N-acetyl-β-glucosaminidase = chitinase (NAG; EC 3.2.1.30), β-glucosidase (β-gluc; EC 3.2.1.21), α-glucosidase (α-gluc; EC 3.2.1.20), β-xylosidase = xylosidase (Xylos; EC 3.2.2.27), acid phosphatase = phosphatase (Phosph; EC 3.1.3.2), arylsulphatase = sulphatase (Aryls; EC 3.1.6.1) and leucine-aminopeptidase = leucine (L.AP; EC 3.4.11.1). The respective substrates were 4-MUF β-D-cellobioside, 4-MUF-N-acetyl-β-glucosaminide, 4-MUF β-D-glucoside, 4-MUF α-D-glucoside, 4-MUF-7-β-D-xyloside, 4-MUF-phosphate, 4-MUF-sulphate and L-leucine-7-amino-4-methylcoumarin (AMC). Before starting the analysis, all soil samples were adjusted at 60% water holding capacity and kept at 25°C for 3 days in the dark. Thus, a suspension of soil was obtained by homogenizing 2 g of each samples with 50 ml sterile water using an Ultra Turrax (IKA, Staufen, Germany) at 9600 rpm for 3 min. Aliquots of 50 μl were withdrawn and dispensed into 96-well black microplates (three analytical replicates per sample per substrate). Fifty microliters of sodium acetate buffer were added at 0.5 M and pH 5.5 for Cell, NAG, β-gluc, α-gluc, Xylos, Phosph and Aryls, whereas of TrisHCl buffer at 0.05 M and pH 7.8 for L.AP. One hundred µl of 1 mM substrate solution was added to all samples, obtaining a final substrate concentration of 500 μM. Plates were kept at 30°C for 30 minutes and then fluorescence (excitation 360 nm; emission 450 nm) was measured with an automated fluorimetric plate-reader (Fluoroskan Ascent, Labsystem GmbH, Frankfurt, Germany) after 0, 30, 60, 120 and 180 minutes (Marinari et al., 2013).

The Synthetic Enzymatic Index (SEI), expressed as sum of all enzymatic activities, was calculated as a synthetic measure of microbial functional capacity. Synthetic enzyme index for the C-cycle (SEIc) was calculated by the sum of the enzymatic activity values of Cell, NAG, β-gluc, α-gluc and Xylos. Microbial functional diversity was assessed by calculating the Shannon diversity index (*H*’) defined as H’ = - ∑ pi * ln pi where pi is the ratio of the activity of a particular enzyme to the sum of all enzymatic activities (Shannon and Weaver, 1949).

Finally, the ecoenzymatic C/N and N/P acquisition activities were measured by the ratios of β-glucosidase/(chitinase+leucine) [β-gluc/(NAG+L.AP)] and (chitinase+leucine)/phosphatase activities [(NAG+L.AP)/Phosph], respectively (Sinsabaugh et al., 2009).

| **TABLE S2 \|** Phylum affiliation, sequence identity, sample origin and taxon of the representative newly generated sequences of Chytridiomycota, Glomeromycota and Mortierellomycota [Internal Trascribed Spacer (ITS1) region] obtained in this study. The taxa were assigned by BLAST against the ITS UNITE database (Kõljalg et al., 2013) and were selected by dynamic threshold values of clustering. The taxon affiliation was used for calculating the relative abundances utilized also for permutational analyses of variance and principal coordinate analyses. | | | | |
| --- | --- | --- | --- | --- |
| **Phylum affiliation** | **Representative sequence code** | **Sample origin** | **OTU name** ^a^ | **Taxon** |
| Chytridiomycota | 10F.127647 | CTN0 15-30 ^b^ | OTU1Spizellomyceta | *Spizellomycetaceae* sp. |
| Chytridiomycota | 10F.134326 | CTN0 15-30 | OTU1Rhizophydiales | *Rhizophydiales* sp. |
| Chytridiomycota | 11F.334948 | CTN0 0-15 | OTU2Rhizophydiales | *Rhizophydiales* sp. |
| Chytridiomycota | 15F.1171599 | MTN200 0-15 | OTU1Chytridiomycota | Chytridiomycota sp. |
| Glomeromycota | 10F.3488 | MTN200 0-15 | OTU6Glomeraceae | *Glomeraceae* sp. |
| Glomeromycota | 10F.138346 | MTN200 0-15 | OTU3Glomeraceae | *Glomeraceae* sp. |
| Glomeromycota | 13F.829262 | MTN200 0-15 | OTU7Glomeraceae | *Glomeraceae* sp. |
| Glomeromycota | 13F.826138 | MTN200 0-15 | OTU1Glomeraceae | *Glomeraceae* sp. |
| Glomeromycota | 13F.836029 | MTN200 0-15 | OTU2Glomeraceae | *Glomeraceae* sp. |
| Glomeromycota | 15F.1187798 | MTN200 0-15 | OTU5Glomeraceae | *Glomeraceae* sp. |
| Glomeromycota | 17F.1586755 | MTN200 0-15 | OTU4Glomeraceae | *Glomeraceae* sp. |
| Glomeromycota | 11F.369970 | MTN200 0-15 | OTU1Claroideo | *Claroideoglomeraceae* sp. |
| Glomeromycota | 1F.3290125 | CT N200 0-15 | OTU1Paraglomero | *Paraglomerales* sp. |
| Mortierellomycota | 10F.124115 | CTN0 15-30 | OTU1Mortierella | *Mortierellaceae* sp. |
| ^a^OTU name: Operational Taxonomic Unit reported in Figures 3 and 4; | | | |  |
| ^b^MTN0 (minimum tillage and 0 kg N ha^-1^); MTN200 (minimum tillage and 200 kg N ha^-1^); CTN0 (conventional tillage and 0 kg N ha^-1^); CTN200 (conventional tillage and 200 kg N ha^-1^); 0-15: 0-15 cm soil depth; 15-30: 15-30 cm soil depth. | | | | |

| **TABLE S3 \|** Effect of tillage and nitrogen fertilization on richness, Shannon index (*H'*) and Simpson index (λ) of soil prokaryotes and fungi at different phylogenetic resolution and at two soil depths, in a wheat-soybean rotation in the Mediterranean area. | | | | | | | |  |
| --- | --- | --- | --- | --- | --- | --- | --- | --- |
|  | **0-15 cm** | | |  | **15-30 cm** | | |  |
|  | **Richness** | ***H'*** | **λ** |  | **Richness** | ***H'*** | **λ** |  |
| *Prokaryotes at class level* | |  |  |  |  |  |  |  |
| MTN0 | 45.33±0.67 ^a^ | 3.30±0.03 a | 0.97±0.00 a |  | 41.67±1.33 | 3.15±0.05 | 0.96±0.00 |  |
| MTN200 | 44.00±0.00 | 3.33±0.01 b | 0.98±0.00 b |  | 41.00±2.00 | 3.11±0.07 | 0.96±0.01 |  |
| CTN0 | 45.00±0.58 | 3.23±0.03 a | 0.97±0.00 a |  | 43.33±0.88 | 3.16±0.05 | 0.96±0.00 |  |
| CTN200 | 45.00±0.58 | 3.36±0.03 b | 0.98±0.00 b |  | 43.67±1.33 | 3.30±0.01 | 0.98±0.00 |  |
| *Prokaryotes at phylum level* | |  |  |  |  |  |  |  |
| MTN0 | 19.67±0.33 | 2.37±0.03 a | 0.91±0.00 a |  | 18.00±0.99 a | 2.25±0.04 a | 0.90±0.01 |  |
| MTN200 | 19.33±0.67 | 2.42±0.01 b | 0.93±0.00 b |  | 16.67±0.67 a | 2.22±0.06 a | 0.90±0.01 |  |
| CTN0 | 19.33±0.88 | 2.31±0.02 a | 0.91±0.00 a |  | 19.00±0.58 b | 2.28±0.04 b | 0.91±0.01 |  |
| CTN200 | 19.33±0.33 | 2.39±0.02 b | 0.92±0.00 b |  | 19.67±0.88 b | 2.39±0.01 b | 0.92±0.00 |  |
| *Fungi at family level* |  |  |  |  |  |  |  |  |
| MTN0 | 55.33±0.88 a | 3.57±0.04 a | 0.98±0.00 a |  | 60.67±0.67 | 3.59±0.03 | 0.98±0.00 a |  |
| MTN200 | 59.33±0.67 b | 3.64±0.02 a | 0.98±0.00 a |  | 54.33±5.24 | 3.57±0.07 | 0.98±0.00 a |  |
| CTN0 | 59.67±1.45 b | 3.71±0.01 b | 0.99±0.00 b |  | 59.00±2.52 | 3.66±0.02 | 0.99±0.00 b |  |
| CTN200 | 56.67±1.67 a | 3.60±0.02 a | 0.98±0.00 a |  | 55.33±2.60 | 3.65±0.02 | 0.99±0.00 b |  |
| *Fungi at phylum level* |  |  |  |  |  |  |  |  |
| MTN0 | 5.00±0.00 | 1.35±0.06 | 0.73±0.02 |  | 5.00±0.00 | 1.41±0.03 | 0.76±0.01 |  |
| MTN200 | 5.00±0.00 | 1.37±0.04 | 0.74±0.02 |  | 5.00±0.00 | 1.36±0.02 | 0.75±0.01 |  |
| CTN0 | 5.00±0.00 | 1.40±0.00 | 0.76±0.00 |  | 5.00±0.00 | 1.36±0.02 | 0.75±0.01 |  |
| CTN200 | 5.00±0.00 | 1.34±0.01 | 0.75±0.01 |  | 5.00±0.00 | 1.39±0.01 | 0.76±0.00 |  |
| ^a^Values are means ± SE of three replicate plots for each treatment. Values in the same column, within each domain (prokaryotes: bacteria and archaea) and kingdom (fungi) at each level of phylogenetic resolution, followed by different letters are statistically different among treatments, according to the two-way ANOVA and the Tukey-B-test as post-hoc test (*P* ≤ 0.05). The values that did not show any statistical difference are reported only by means ± SE. | | | | | | | |  |

| **TABLE S4 \|** Permutational analyses of variance (PERMANOVAs) on the long-term effect of tillage and nitrogen fertilization on soil prokaryotic and fungal diversity at different phylogenetic resolution and at two soil depths in a wheat-soybean rotation in the Mediterranean area. | | | | | | | |
| --- | --- | --- | --- | --- | --- | --- | --- |
|  | **0-15 cm** | | |  | **15-30 cm** | | |
|  | **Total df** | **Pseudo-F** | ***P* (perm)** |  | **Total df** | **Pseudo-F** | ***P* (perm)** |
| *Prokaryotes at class level* | |  |  |  |  |  |  |
| TIL ^a^ | 1 | 1.44 | 0.265 ^b^ |  | 1 | 2.90 | 0.075 |
| N fert | 1 | 6.13 | **0.014** |  | 1 | 1.04 | 0.366 |
| TIL x N fert | 1 | 6.83 | **0.010** |  | 1 | 1.69 | 0.205 |
| *PERMDISP* |  |  |  |  |  |  |  |
| TIL |  |  | **0.012** |  |  |  | - |
| N fert |  |  | 0.791 |  |  |  | - |
| *Prokaryotes at phylum level* | | |  |  |  |  |  |
| TIL | 1 | 2.33 | 0.125 |  | 1 | 2.02 | 0.174 |
| N fert | 1 | 4.85 | **0.034** |  | 1 | 0.72 | 0.499 |
| TIL x N fert | 1 | 3.36 | 0.055 |  | 1 | 1.49 | 0.275 |
| *PERMDISP* |  |  |  |  |  |  |  |
| TIL |  |  | - |  |  |  | - |
| N fert |  |  | 0.532 |  |  |  | - |
| *Fungi at family level* | |  |  |  |  |  |  |
| TIL | 1 | 7.74 | **0.002** |  | 1 | 5.29 | **0.004** |
| N fert | 1 | 5.90 | **0.003** |  | 1 | 3.62 | **0.011** |
| TIL x N fert | 1 | 5.52 | **0.003** |  | 1 | 3.40 | **0.004** |
| *PERMDISP* |  |  |  |  |  |  |  |
| TIL |  |  | **0.040** |  |  |  | 0.865 |
| N fert |  |  | 0.412 |  |  |  | **0.044** |
| *Fungi at phylum level* | |  |  |  |  |  |  |
| TIL | 1 | 23.60 | **0.003** |  | 1 | 3.36 | **0.025** |
| N fert | 1 | 4.36 | **0.018** |  | 1 | 0.45 | 0.706 |
| TIL x N fert | 1 | 9.19 | **0.002** |  | 1 | 0.71 | 0.548 |
| *PERMDISP* |  |  |  |  |  |  |  |
| TIL |  |  | **0.016** |  |  |  | 0.594 |
| N fert |  |  | **0.005** |  |  |  | - |
| ^a^PERMANOVAs were performed following a split-plot design with tillage (TIL) as main-plot factor and nitrogen fertilization (N fert) as subplot factor and with three replicate plots per treatment: TIL (minimum tillage and conventional tillage) and N fert (0 kg N ha^-1^ and 200 kg N ha^-1^); | | | | | | | |
| ^b^In bold statistically significant values (*P* ≤ 0.05). | | | | | | | |

| **TABLE S5 \|** Effect of soil depth (0-15 *vs* 15-30 cm) on richness, Shannon index (*H'*) and Simpson index (λ) of soil prokaryotes and fungi at different phylogenetic resolution in a wheat-soybean rotation in the Mediterranean area. Tillage and nitrogen fertilization are used as covariates. | | | |
| --- | --- | --- | --- |
|  | **Richness** | ***H'*** | **λ** |
| *Prokaryotes at class level* | |  |  |
| 0-15 cm | 44.83±0.27 ^a^ b | 3.31±0.02 b | 0.97±0.00 b |
| 15-30 cm | 42.42±0.70 a | 3.20±0.03 a | 0.96±0.00 a |
| *Prokaryotes at phylum level* | |  |  |
| 0-15 cm | 19.42±0.26 | 2.37±0.01 b | 0.92±0.00 b |
| 15-30 cm | 18.33±0.48 | 2.29±0.03 a | 0.91±0.00 a |
| *Fungi at family level* |  |  |  |
| 0-15 cm | 57.75±0.76 | 3.60±0.02 | 0.98±0.00 |
| 15-30 cm | 57.33±1.57 | 3.62±0.02 | 0.98±0.00 |
| *Fungi at phylum level* |  |  |  |
| 0-15 cm | 5.00±0.00 | 1.37±0.01 | 0.74±0.01 |
| 15-30 cm | 5.00±0.00 | 1.38±0.01 | 0.75±0.00 |
| ^a^Values are means ± SE of three replicate plots for each treatment. Values in the same column, within each domain (prokaryotes: bacteria and archaea) and kingdom (fungi) at each level of phylogenetic resolution, followed by different letters are statistically different between soil depths, according to one-way ANOVA and the Tukey-B-test as post-hoc test (*P* ≤ 0.05). The values that did not show any statistical difference are reported by means ± SE. | | | |

| **TABLE S6 \|** Permutational analyses of variance (PERMANOVAs) on the effect of soil depth (0-15 *vs* 15-30 cm) on soil prokaryotic and fungal diversity at different phylogenetic resolution in a wheat-soybean rotation in the Mediterranean area. Tillage and nitrogen fertilization were used as covariates. | | | |
| --- | --- | --- | --- |
|  | **0-15 *vs* 15-30 cm** | | |
|  | **Total df** | **Pseudo-F** | ***P* (perm)** |
| *Prokaryotes at class level* | |  |  |
| Soil depth ^a^ | 1 | 7.09 | **0.001** ^b^ |
| TIL | 1 | 2.98 | 0.065 |
| N fert | 1 | 3.36 | **0.040** |
| *PERMDISP* |  |  |  |
| Soil depth |  |  | 0.486 |
| *Prokaryotes at phylum level* | |  |  |
| Soil depth | 1 | 3.37 | **0.035** |
| TIL | 1 | 3.34 | **0.037** |
| N fert | 1 | 3.66 | **0.033** |
| *PERMDISP* |  |  |  |
| Soil depth |  |  | 0.350 |
| *Fungi at family level* | |  |  |
| Soil depth | 1 | 4.97 | **0.001** |
| TIL | 1 | 3.80 | **0.001** |
| N fert | 1 | 3.69 | **0.001** |
| *PERMDISP* |  |  |  |
| Soil depth |  |  | 0.301 |
| *Fungi at phylum level* | |  |  |
| Soil depth | 1 | 1.83 | 0.154 |
| TIL | 1 | 0.26 | 0.799 |
| N fert | 1 | 0.06 | 0.929 |
| *PERMDISP* |  |  |  |
| Soil depth |  |  | - |
| ^a^PERMANOVAs were performed using soil depth as fixed factor, tillage (TIL) and nitrogen fertilization (N fert) as covariates and 12 replicates plots per soil depth. TIL: minimum tillage and conventional tillage; N fert: 0 kg N ha^-1^ and 200 kg N ha^-1^; | | | |
| ^b^In bold statistically significant values (*P* ≤ 0.05). | | | |


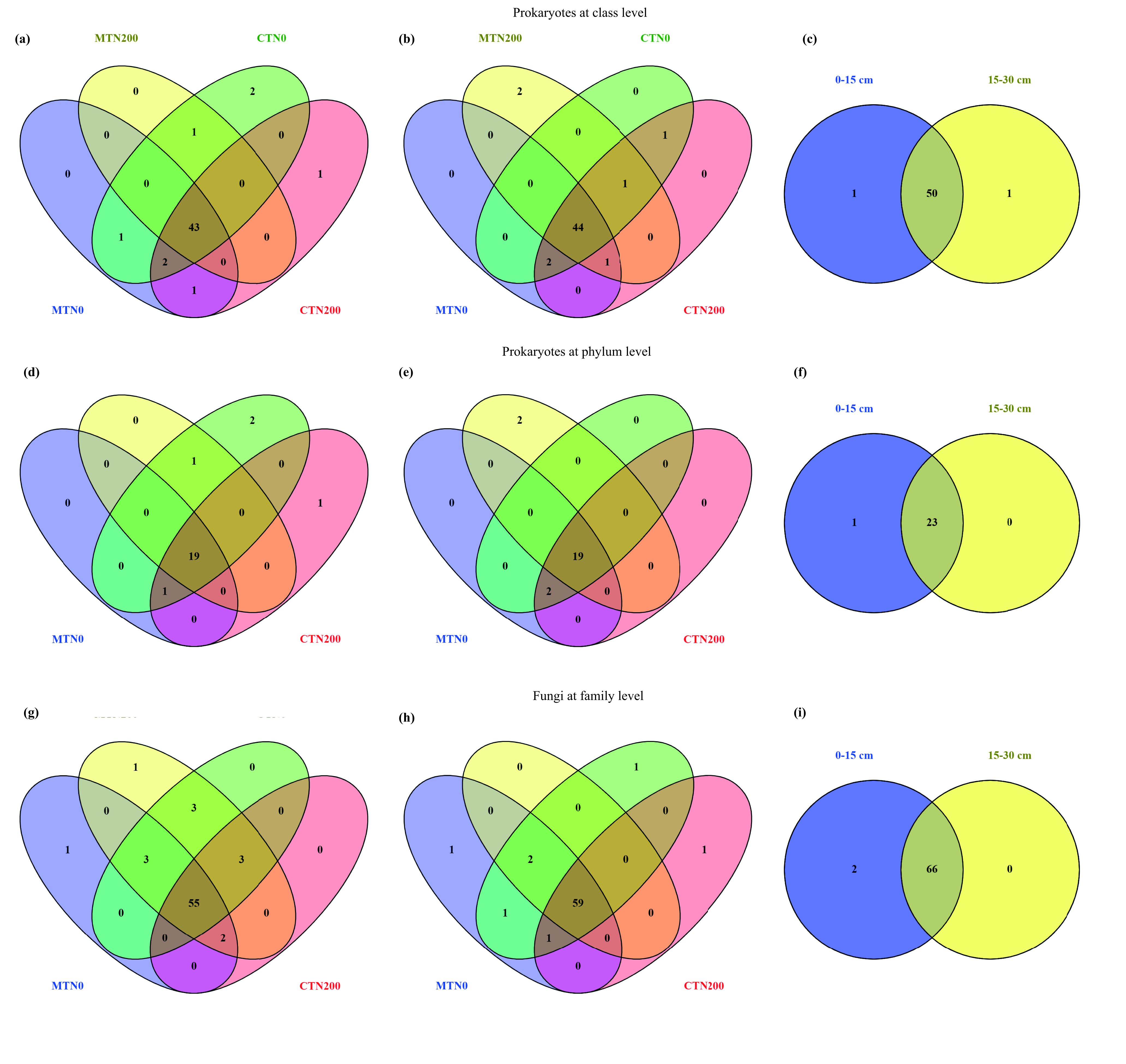
**FIGURE S1 |** Venn diagrams showing the number of prokaryotic and fungal taxa retrieved in soil unique to and shared among treatments: MTN0 (minimum tillage and 0 kg N ha^-1^); MTN200 (minimum tillage and 200 kg N ha^-1^); CTN0 (conventional tillage and 0 kg N ha^-1^); CTN200 (conventional tillage and 200 kg N ha^-1^); and between soil depths: 0-15 cm and 15-30 cm soil depth. Among treatments: prokaryotes at class level at 0-15 and 15-30 cm soil depth **(a,b)**; prokaryotes at phylum level at 0-15 and 15-30 cm soil depth **(d,e)**; fungi at family level at 0-15 and 15-30 cm soil depth **(g,h)**. Between soil depth: prokaryotes at class and phylum level **(c,f)** and fungi at family level **(i)**.

**References**

Apprill, A., McNally, S., Parsons, R., and Weber, L. (2015). Minor revision to V4 region SSU rRNA 806R gene primer greatly increases detection of sar11 bacterioplankton. Aquat. Microb. Ecol. 75, 129-137. doi:10.3354/ame01753.

Blake, G. R., and Hartge, K. H. (1986). Bulk Density 1. Methods of Soil Analysis: Part 1 - Physical and Mineralogical Methods, 363-375.

Bremner, J. M., and Mulvaney, C. S. (1982). Nitrogen - Total 1. Methods of soil analysis. Part 2. Chemical and microbiological properties, 595-624.

Gardes, M., and Bruns, T. D. (1993). ITS primers with enhanced specificity for basidiomycetes application to the identification of mycorrhizae and rusts. *Mol. Ecol.* 2, 113-118. doi:10.1111/j.1365-294X.1993.tb00005.x.

Goldman, E., and Jacobs, R. (1961). Determination of nitrates by ultraviolet absorption. *J. Am. Water Works Assoc.* 53, 187-191.

Ihrmark, K., Bödeker, I., Cruz-Martinez, K., Friberg, H., Kubartova, A., Schenck, J., et al. (2012). New primers to amplify the fungal ITS2 region–evaluation by 454-sequencing of artificial and natural communities. FEMS Microbiol. Ecol., 82(3), 666-677. doi:10.1111/j.1574-6941.2012.01437.x.

Kõljalg, U., Nilsson, R. H., Abarenkov, K., Tedersoo, L., Taylor, A. F., Bahram, M., et al. (2013). Towards a unified paradigm for sequence‐based identification of fungi. Mol. Ecol. 22, 5271-5277. doi:10.1111/mec.12481.

Marinari, S., Bonifacio, E., Moscatelli, M. C., Falsone, G., Antisari, L. V., and Vianello, G. (2013). Soil development and microbial functional diversity: proposal for a methodological approach. *Geoderma* 192, 437-445. doi:10.1016/j.geoderma.2012.08.023.

Marx, M. C., Wood, M., and Jarvis, S. C. (2001). A microplate fluorimetric assay for the study of enzyme diversity in soils. *Soil Biol. Biochem*. 33, 1633-1640. doi:10.1016/S0038-0717(01)00079-7.

Olsen, S. R., Sommers, L. E., and Page, A. L. (1982). Methods of soil analysis. Part 2. Chemical and microbiological properties of Phosphorus. *ASA Monograph* 9, 403-430.

Parada, A. E., Needham, D. M., and Fuhrman, J. A. (2016). Every base matters: assessing small subunit rRNA primers for marine microbiomes with mock communities, time series and global field samples. *Environ. Microbiol.*, 18(5), 1403-1414. doi:10.1111/1462-2920.13023

Shannon, C. E., and Weaver, W. (1949). The Mathematical Theory of Communication. Urbana, IL: University of Illinois Press.

Sinsabaugh, R. L., Hill, B. H., and Shah, J. J. F. (2009). Ecoenzymatic stoichiometry of microbial organic nutrient acquisition in soil and sediment. *Nature* 462, 795-798. doi:10.1038/nature08632.

Vepsäläinen, M., Kukkonen, S., Vestberg, M., Sirviö, H., and Niemi, R. M. (2001). Application of soil enzyme activity test kit in a field experiment. *Soil Biol. Biochem*. 33, 1665-1672. doi:10.1016/S0038-0717(01)00087-6.

White, T. J., Bruns, T., Lee, S. J. W. T., and Taylor, J. L. (1990). “Amplification and direct sequencing of fungal ribosomal RNA genes for phylogenetics”, in PCR protocols: a guide to methods and applications 18, 315-322.
